# Medicinal plants mediated the green synthesis of silver nanoparticles and their biomedical applications

**DOI:** 10.1049/nbt2.12078

**Published:** 2022-04-15

**Authors:** Haajira Beevi Habeeb Rahuman, Ranjithkumar Dhandapani, Santhoshini Narayanan, Velmurugan Palanivel, Ragul Paramasivam, Ramalakshmi Subbarayalu, Sathiamoorthi Thangavelu, Saravanan Muthupandian

**Affiliations:** ^1^ Medical Microbiology Unit Department of Microbiology Alagappa University Karaikudi Tamilnadu India; ^2^ Chimertech Private Limited Chennai Tamilnadu India; ^3^ Centre for Materials Engineering and Regenerative Medicine Bharath Institute of Higher Education and Research Chennai Tamilnadu India; ^4^ Division of Biomedical Sciences College of Health Sciences School of Medicine Mekelle Ethiopia; ^5^ AMR and Nanotherapeutics Laboratory Department of Pharmacology Saveetha Dental College and Hospital Saveetha Institute of Medical and Technical Sciences (SIMATS) Chennai Tamilnadu India

**Keywords:** antimicrobial, eco‐friendly, green synthesis, silver nanoparticles

## Abstract

The alarming effect of antibiotic resistance prompted the search for alternative medicine to resolve the microbial resistance conflict. Over the last two decades, scientists have become increasingly interested in metallic nanoparticles to discover their new dimensions. Green nano synthesis is a rapidly expanding field of interest in nanotechnology due to its feasibility, low toxicity, eco‐friendly nature, and long‐term viability. Some plants have long been used in medicine because they contain a variety of bioactive compounds. Silver has long been known for its antibacterial properties. Silver nanoparticles have taken a special place among other metal nanoparticles. Silver nanotechnology has a big impact on medical applications like bio‐coating, novel antimicrobial agents, and drug delivery systems. This review aims to provide a comprehensive understanding of the pharmaceutical qualities of medicinal plants, as well as a convenient guideline for plant‐based silver nanoparticles and their antimicrobial activity.

## 1 INTRODUCTION

**FIGURE 1:**
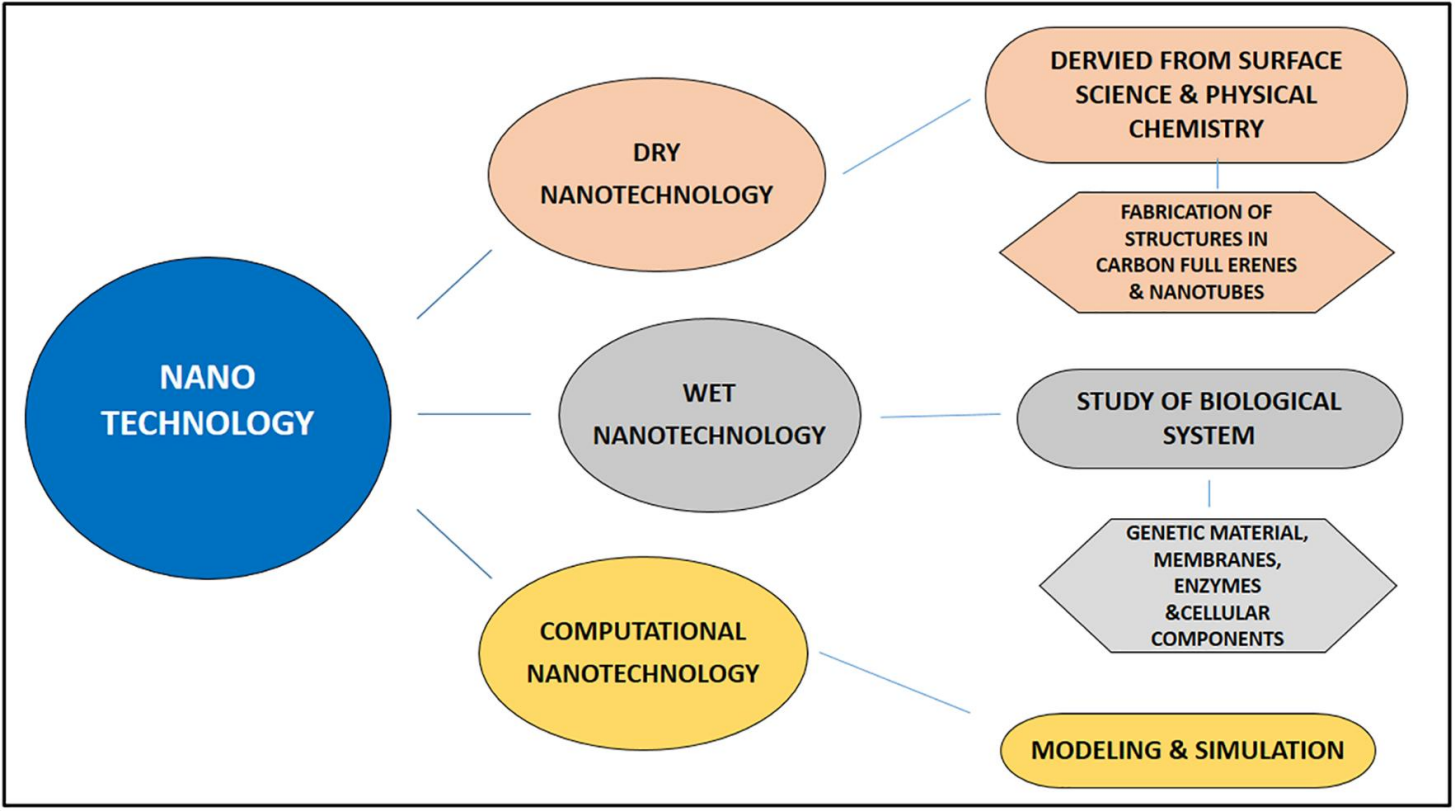
Major three disciplines of nanotechnology dry nanotechnology, wet nanotechnology, and computational nanotechnology

Nanotechnology is a growing industry that can be used to create nanoscale structures. Nanoproducts are concerned with the approach and synthesis of particles with a diameter ranging from 1 to 100 nm. In living beings, nanotechnology is a combination of wet, dry, and computerised nanotechnology. Wet nanotechnology encompasses biological agents such as membranes, organs, and enzymes. Dry nanotechnology deals with surface science, physical, chemical properties, and the production of inorganic materials such as silicon and carbon. Modelling and simulation of complex nanometre‐scale structures are part of computational nanotechnology [1]. Figure 1 depicts how these three disciplines were intertwined for optimal functionality. Nanoscale compounds can be distinguished by specific properties, opening up a wide range of applications, as well as the extension of science nanoscale discipline and research opportunities. It is used in a variety of industries, including pharmaceuticals, diagnostics, consumer goods, and supplements in healthcare products, targeted drug delivery systems, growth inhibitors of biofilm formation, biosensors, bioremediation, and electronics [2]. Nanoparticles (NPs) are a broad class of materials made up of particles with a minimum size of 100 nm [3]. NPs are divided into three layers because they are not simple molecules: (1) The surface layer, which can be stabilised with a variety of unique compounds, metal ions, emulsifiers, and polymers; (2) The shell layer, which is chemically and physically distinct from the core; and (3) The core, which could be the NPs’ central element [4]. NPs optical properties are influenced by their size, resulting in different colouration caused by the absorption in the visible region. Their size, shape, and structure have an impact on their reactivity, durability, and other properties [5]. Heavy metal NPs such as lead, Mercury, and tin are so hard and strong that their decomposition is difficult, posing a variety of environmental hazards [5]. Because of the added focus on the combination of nanotechnology and biotechnology, there is indeed a greater emphasis on the development of medical materials [6]. Certain herbs have been used in traditional medicine for centuries. The medicinal plants and their parts contain a wide range of beneficial elements, including bioactive compounds. These medicinal herbs are high in alkaloids, phenolics, and tannins. Such biologically active compounds in plants could indeed speed up the conversion of metal ions into biologically active nanoparticles in an environmentally friendly standard biosynthesis pathway [7]. Plant components such as fruits, leaves, stems, seeds, flowers, roots, bark, rhizome, and fruit peel are used in the production of various types of nanoparticles [8].

Silver nanoparticles (AgNPs) have recently been thoroughly investigated for their physical, chemical, and biological properties, which vary in scale, form, function, crystallinity, and structures. Several studies are well underway to incorporate AgNPs into clinical and industrial technologies as well as drug applications [9, 10]. Silver has been described as ‘dynamic’ because of its ability to exert excellent potential for biological uses, including antifungal, antibacterial, antiviral, anti‐infectious, wound healing, and anti‐inflammatory properties at low concentrations [11, 12]. Silver is a non‐toxic inorganic antibacterial agent capable of eliminating approximately 650 different types of disease‐causing microorganisms [13, 14]. Even though AgNPs have become more extensively used in medicine and everyday life, there is a lack of detailed biological and toxicological data [15, 16]. AgNPs are increasingly used in a wide variety of commercial products all over the world. Living creatures are exposed to nanoparticles either actively or passively, raising the concerns regarding their toxicity. Therefore, the requirement of the appropriate usage of nanoparticles in various biological purposes must be defined. However, there is indeed a paucity of verified evidence on the impact of AgNPs on the environment, animals, humans as well as the possible concerns associated with their short‐ and long‐term hazardous consequences. AgNPs are also used in the garment industry and in wastewater treatment. Scientists are intrigued by the green production of AgNPs wide applications. AgNPs green synthesis had already been shown to be a promising and environmentally friendly method [17]. The green synthesis method makes the mass production of nanoparticles safer and less expensive [7]. In this review, we discussed the formation of medically valuable AgNPs and their characterisation, various biomedical features of AgNPs with their mechanism of action and also the potential hazardous effects of AgNPs. The pilot project of plant‐mediated nanoparticle synthesis and their applications has special interest. As a result, the review not only gives a complete overview of the applications and the present situation of the plant‐mediated AgNPs synthesis, but it also gives insight on a highly promising research area.

## 2 APPROACHES OF NANOPARTICLE PRODUCTION

**FIGURE 2:**
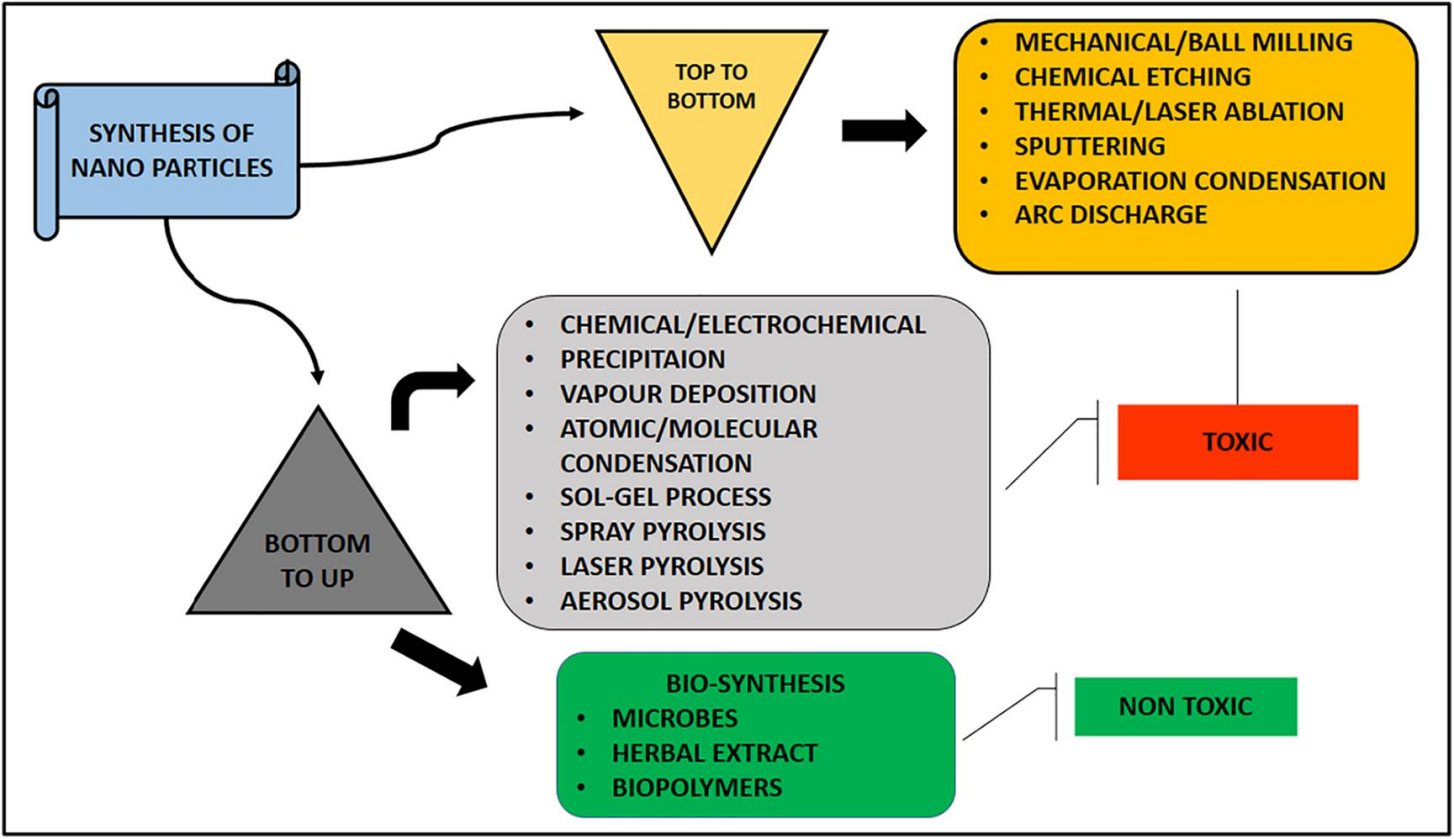
Two approaches of formation of metallic nanoparticles Top to bottom and Bottom to up

The man‐made approaches to AgNPs synthesis are currently classified as physical, chemical, and biological. The biological synthesis of AgNPs exhibits desirable properties such as high yield, solubility, and stability, whereas physical and chemical synthesis of AgNPs appear to be more labour‐intensive and riskier [18, 19]. As illustrated in Figure 2, there are two approaches used in the formulation of metallic nanoparticles: a top‐bottom and a bottom‐up approach. In the top‐bottom approach, nanoparticles are synthesised through either of these methods, which includes mechanical/ball milling, chemical etching, thermal/laser ablation, sputtering, evaporation condensation, and arc discharge [20, 21]. The bottom‐top approach necessitates starting with molecules or atoms to create nanoparticles through chemical, precipitation, vapour deposition, atomic/molecular condensation, sol‐gel process, and spray/laser/aerosol pyrolysis methods. In physical methods, nanoparticles are generated using the evaporation–condensation process in a tubular furnace at atmospheric pressure. The benefits of physical methods include speed, the use of radiation as a reducing agent, and the absence of hazardous chemicals. However, the disadvantages include poor productivity, high power consumption, solvent contamination, and the inability to achieve a homogenous distribution. The chemical synthesis methods are classified as chemical reduction, electrochemical, and pyrolysis [10]. To reduce the metal ion, various organic and inorganic agents such as NaBH4, ascorbate, tollens reagent, N‐dimethyl formamide (DMF), and polyethylene glycol have been used in aqueous and non‐aqueous solutions [22]. The greater benefit of this chemical reduction is that a huge proportion of nanoparticles are synthesised in a shorter period. This method of synthesis results in the formation of non‐eco‐friendly byproducts, which is the primary reason to opt the green route approach to produce nanoparticles.

Biosynthesis of nanoparticles involves microbes, herbal extracts, and biopolymers. Since ancient times, the potential of plants as a source of drugs and herbal remedies has been extensively investigated. Pharmacological screening of natural source compounds has yielded a plethora of therapeutic agents, and it is estimated that approximately 8000 natural antibiotics have been isolated, characterised, synthesised, and discovered to have potent biological activities all over the world. In general, nanoparticles are created through a variety of chemical and physical processes that are both costly and potentially hazardous to the environment, as they involve the use of harmful and dangerous chemicals that are responsible for a variety of potential consequences. In the biological synthesis method, bio absorption of metals by gram‐positive and gram‐negative bacteria provided evidence for the synthesis of nanoparticles. Moreover, the synthesised nanomaterials were just aggregating rather than nanoparticles. Many studies have been reported on the biological formulation of AgNPs using microbes such as bacteria, fungi, and plants [18]. Bacterial species such as *Lactobacillus sp., Thermomonospora sp., P. stutzeri, Aspergillus flavus, Torulopsis sp., Fusarium oxysporum,* and *Verticillium sp.* are used to synthesise metal nanoparticles and their activities have been thoroughly investigated [23, 24]. Several biomolecules, including biopolymers, starch, fibrinolytic enzyme, and amino acids were also used. Three factors determine the biological synthesis of nanoparticles: (1) the solvent; (2) the reducing agent; and (3) the non‐toxic element. The existence of amino acids, proteins, or secondary metabolites existing in the synthesis method removes the additional step to prevent particle agglomeration.

The use of biomolecules for the AgNPs synthesis is an eco‐friendly and pollution‐free method. Biological methods appear to provide controlled particle size and shape, which is critical for a wide range of medical applications. We could indeed regulate the shape, size, and monodispersed nanoparticles by using bacterial protein or plant extracts as reducing agents. Other benefits of biological methods include the availability of a diverse variety of natural resources, a reduced time necessity, high density, and stability. The smaller size and shortened nanoparticles appear to be more effective and have superior properties in terms of size and shape. Although studies have successfully synthesised AgNPs of various shapes and sizes, there are some drawbacks. Compared to chemical methods, biological synthesis methods lead to the increased regulation of the size, shape, and dispersion of the generated nanoparticles; and the optimization of synthesis methods, which includes the number of precursors, pH, temperature, and the amount of reducing and stabilising elements.

## 3 FORMULATION OF SILVER NANOPARTICLES

**FIGURE 3:**
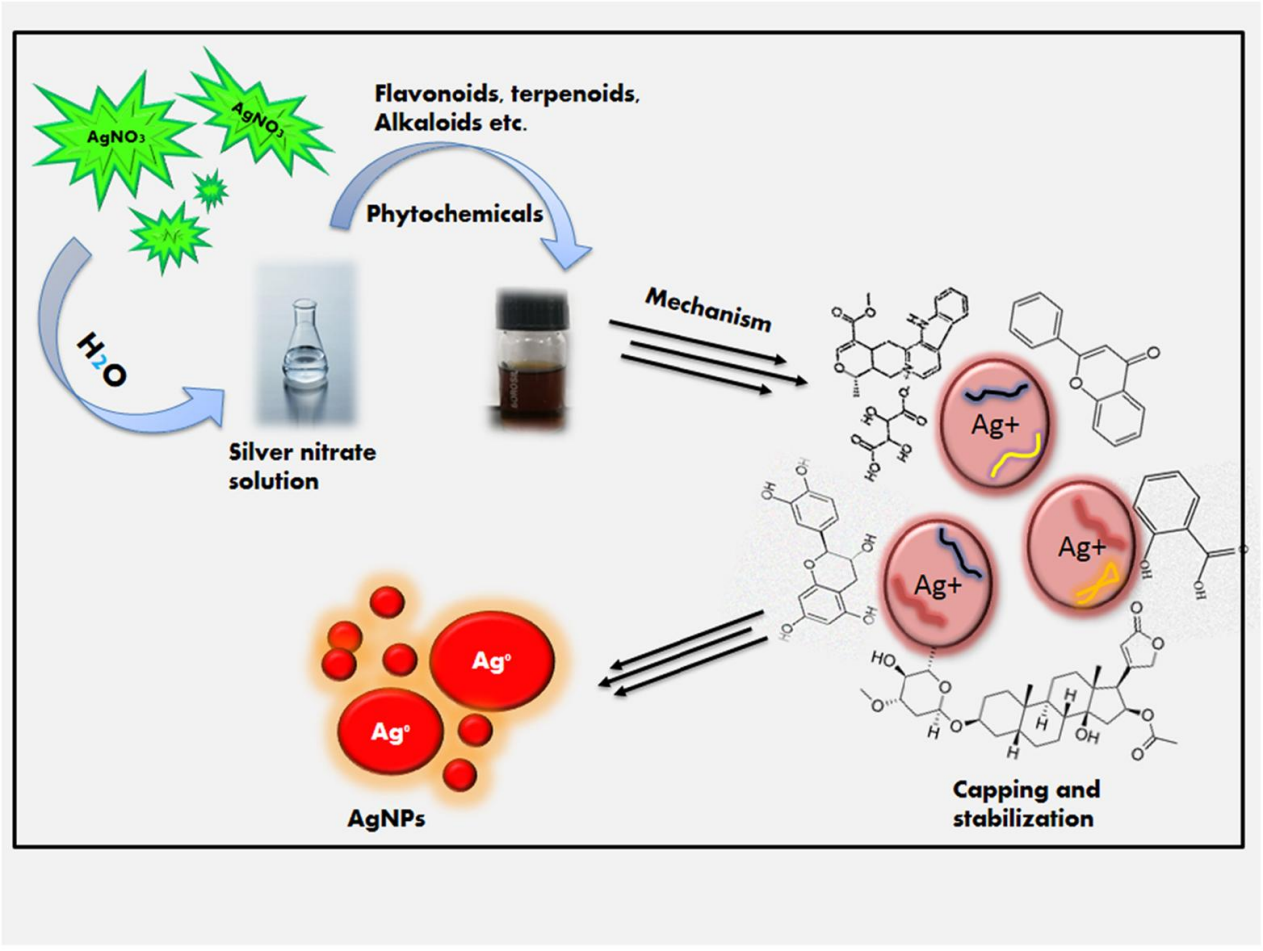
Synthesis of silver nanoparticles (AgNPs) using plant extract with silver nitrate (AgNO3) solution

AgNPs are considered important among various noble metal nanoparticles due to their noticeable characteristics, which include favourable conductivity, stability, and potential antimicrobial activity. Green synthesised AgNPs are well recognized for their biomedical and pharmaceutical applications, are environmentally friendly, cost effective, easily scalable, and produce high yields than chemically produced AgNPs [25, 26, 27]. Many phytochemicals are found in medicinal plants. The phytochemicals play an important role in the formulation of nanoparticles [28]. Nanoparticles (NPs) bound with natural compounds have proven to be more beneficial and effective than traditional herbal drugs [29]. The plant crude extract was prepared using various methods, including soxhlet extraction, a solvent extraction method using ethanol, and methanol, and a decoction extraction method using an Erlenmeyer flask [30, 31, 32]. These natural compounds were added to the silver nitrate (AgNO_3_) solution and these compounds act as a capping and stabilising agent that result in the reduction of pure Ag(I) to Ag(0). During the green synthesis of AgNPs, the primary goal is to enhance safety and reliability, thereby avoiding the ecological and economic damage of hazardous raw resources. The proportionate amount of plant extract and metal ion, synthesis time, temperature, and the reaction pH could significantly determine the origin yield, quality, and features of the generated AgNPs [33]. Although different plants and their components might involve various biomolecules that could act as reducing and stabilising agents during synthesis, the selection of plants and their components might well be significant in biosynthesis. These phytomolecules could also have an impact on the AgNPs surface properties. The synthesis of AgNPs using a plant extract was illustrated in Figure 3.

The production of nanoparticles from living sources is far safer than that of chemical or physical methods. The production of stable AgNPs using a biological method employing microorganisms seems to be beneficial over other methods. Other biological molecules used in the production of AgNPs include biopolymers (lignin, chitosan, polypeptides, alginate, and cellulose), starch, enzymes, and amino acids. It is hypothesised that the biomolecules contain particularly secondary metabolites (vitamins, polysaccharides, amino acids, proteins, enzymes, polyphenolics, flavonoids, terpenoids, alkaloids, phenolic acids, alcohol, antioxidant, alkynes, allylic benzenes, ascorbic acids, anthraquinones, benzoates, alcoholic compounds, amide, amino acid residues, caffeoyl, carbohydrates, carotenes, phenolic compounds, steroids, sugars, tannins, saponins, triterpenoids, glycosides, leucocyanidin, iridoids, and catechic tannins) that could reduce the Ag ions [34]. Functional groups, such as carboxyl (COOH), observed in glutamine and aspartic byproducts, as well as the OH group of tyrosine, are believed to be responsible for Ag ion stabilisation and the formation of small polydisperse Ag nanoplates. Nowadays, the use of microbial cells for the synthesis of metal NPs has proven to be an excellent method. Microbial cells demonstrate to be outstanding bio factories for AgNPs production. Bacteria are recommended over other microbes for nanoparticles production because they could be cultivated in a controlled condition. Microbes could respond to higher metal levels and have the ability to reduce inorganic compounds into nanoparticles through the use of extracellular and intracellular synthesis. Microbes capture metal ions from their surroundings and transform them into an elemental state mostly through enzymatic reduction.

**FIGURE 4:**
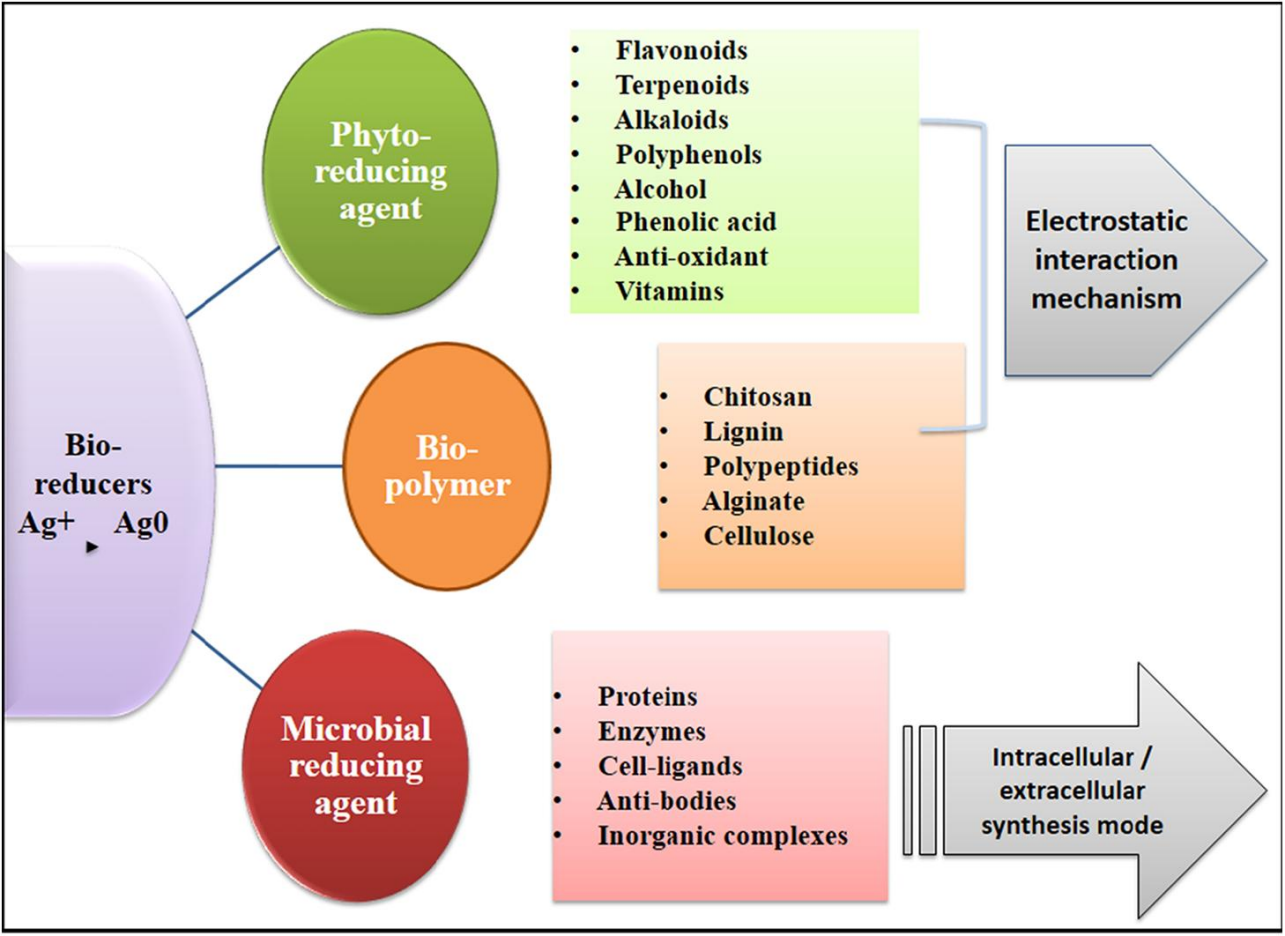
Extracellular and intracellular synthesis of silver nanoparticles (AgNPs)

Microorganism‐mediated synthesis of AgNPs is indeed not a feasible method for industries due to its highly aseptic condition and cell culture maintenance for the large production of NPs [35]. Figure 4 depicts the division of bacterial synthesis into two groups: extracellular synthesis and intracellular synthesis. Metal ions are trapped at the cell surface by the extracellular synthesis of AgNPs. The microbial cell containing broth is collected by centrifugation, and the supernatant containing microbial enzymes is used in the synthesis of NPs. Metal ions are reduced in a cell‐free supernatant, resulting in the formation of nanoparticles. This extracellular synthesis of AgNPs is cost‐effective and involves simple downstream processing [36]. Extracellular synthesis is more consistent than intracellular synthesis [30, 37]. In the intracellular fabrication method, the cellular mechanism of microbial cells is used for the synthesis of NPs. The microbial biomass and metal solution would then be cultured at the desired incubation conditions until a particular colour change is detected. When the pale‐yellow colour of the solution changes to brownish colour, it indicates the formation of AgNPs. Metal ions are locked within the cell wall of microbial cells and the enzymes produced by the microbes reduce these metal ions within the cell wall, resulting in the generation of nanoclusters, and the nanoparticles eventually dispersed from the cell wall to the aqueous solution.

## 4 FACTORS INFLUENCING THE PLANT‐MEDIATED SYNTHESIS OF SILVER NANOPARTICLES

The key problems in AgNPs biosynthesis are the regulation of crystalline structure, shape, size, and size distribution, and the principle causes impacting these characteristics are detailed here.

### 4.1 Effect of metal ion concentration (silver nitrate)

AgNO_3_ was employed as a precursor in most of the plant‐mediated synthesis of AgNPs and its concentration had a significant effect on the particle size of the nanoparticles. For instance, Rahuman et al. 2021 [38] found that optimum yield of AgNPs was obtained in 1.25 mM concentration of silver nitrate and it also showed a strong intense peak at this concentration with the surface plasmon resonance (SPR) peak at 410 nm. AgNPs synthesised using *Shorea robusta* leaf extract revealed the optimum concentration of silver nitrate to be 1.25 mM for the bioreduction process and also shows that a wider SPR peak was observed in lower concentrations, which was due to the occurrence of larger sized nanoparticles. In addition, a narrow SPR peak in higher concentrations represent the decreased nanoparticle size [39].

### 4.2 Effect of substrate concentration (plant extract)

The biomolecules present in the extracts are essential for both the reduction of silver and stability of generated AgNPs. It is widely known that raising biomolecules in the reaction could boost the AgNPs production and expand the size and modify the structure to an optimal condition. The absorption spectrum narrowed when the leaf extract concentration was increased indicating a decrease in the nanoparticle size. Rahuman et al. 2021 [38] study shows that at 1.25 ml of leaf extract concentration, the AgNPs reaction takes place quickly with the SPR peak at 410 nm.

### 4.3 Effect of Silver nanoparticles’ production time

The incubation period for a certain plant extract has a substantial impact on the size, shape, and characteristics of green synthesised AgNPs. Reduced nanoparticle production and size variation were seen when the optimal incubation duration was not implemented. AgNPs size was increased with an increase in the reaction time, which might be due to the agglomeration of colloidal AgNPs [40]. It is one of the most widely used methods to verify the production and stabilisation of AgNPs. The plant biomolecules such as flavans, flavanol, and flavanonol present in the *Shorea robusta* leaf extract is responsible for the reduction of silver ions within 20 min [39]. Rahuman et al. 2021 presented that the presence of alkaloid, flavonoids, and terpenoids present in the *Carissa carandas* leaf extract is responsible for the reduction and stabilisation of AgNPs, and the greatest production of AgNPs was obtained within 20 min [38].

### 4.4 Effect of pH

The pH of the liquid media has a significant influence on the size, shape, and production of the plant‐mediated AgNPs. At low pH, silver interacts with the amino and sulfhydryl groups and these positive ions reduce Ag^+^ to Ag^0^. Because of the existence of positively charged functional groups, the reduction was mostly accomplished through ionic bonding and biomolecules, which was promoted at lower pH. A large number of biomolecules bind with the silver nanoparticles that subsequently result in agglomeration and larger sized AgNPs. At pH 8 of the reaction mixture, small‐sized AgNPs were observed [41]. At higher pH, quick bio‐reduction and high dispersion of AgNPs with negative zeta potential was observed and yielded a larger particle size [42].

## 5 ROLE OF PHYTOMOLECULES IN SILVER NANOPARTICLES FORMATION

Plants are free of harmful chemicals and contain natural capping agents, which could enable a greener platform for nanoparticle manufacturing. Moreover, plant extracts lower the expenses caused by microbe nanoparticle synthesis, which involves microorganism isolation, culture maintenance and production in a sterile environment. The antibacterial actions of AgNPs were related to the size, shape, and stabilising agents of nanoparticles. Plant extracts operate as a reducing and stabilising agent in the production of antimicrobial nanoparticles, enabling cost‐effective and environment‐friendly operations than traditional physical and chemical approaches. Water soluble phytoconstituents such as flavones, quinones, and organic acids (oxalic, malic, and tartaric) that are available in plants could have contributed towards an instantaneous reduction of silver ions in the synthesis process. Silver reduction achieved through phytoconstituents (flavonoids or other polyphenols) existing in plants might well be viewed as an important progress in this path. Plant‐mediated biosynthesis pathways for the production of AgNPs are faster and more repeatable. Biosynthesis of AgNPs involves the carbonyl and hydroxyl groups of flavonoids, which perform a crucial function in the reduction of silver ions by chelating metal ions with the flavonoids, where the charge transport and electrostatic connection between the OH group of flavonoids and silver ions are essential for the metabolic action of the reduction process. Three distinct flavonoids, namely Flavon‐3‐ol, Flavon‐4‐ol, and Flavon‐3,4‐diol worked as a reducing or stabilising agent for the reduction of Ag^+^ to Ag^0^ [39, 43].

Although biomolecules involved in a process incorporating concurrent reducing and capping that were hypothesised in all these investigations, none of them could definitively determine the phytochemicals engaged in each function. While the majority of review publications on green synthesised AgNPs focussed solely on the application's effectiveness, very few addressed the potential challenges and limitations of this process. The fundamental shortcoming of various research studies in this field is that the findings are sometimes conflicting, and there are no general themes observed. This is because, to a considerable extent, the findings differ substantially due to the various plant extracts and the phytochemicals vary from plant to plant. Additionally, the size and morphology of AgNPs are controlled by certain phytochemicals and background chemistry. To clarify the mechanism, it is necessary to identify the exact phytochemicals participating in the AgNPs production. But in attempting to understand the correlations between production, morphology, and plant extract compounds, there is a lack of appropriate plant extract characterisation in accordance with the common phytoconstituents, which is a huge difficulty. Despite claims that green synthesised AgNPs is more environmentally friendly than the chemical and physical method of nanoparticle synthesis, investigations are unable to demonstrate this due to a major lack of strong scientific data. The claim of environmental friendliness is predicted mostly on the amount of hazardous compounds replaced by non‐toxic substitutes in the biosynthesis, the total energy conserved, and the projected ecological and economic impacts [44]. Commercial AgNPs are synthesised under strict guidelines to ensure quality in terms of size homogeneity and surface characteristics, both are critical for ensuring better performance. Moreover, the form and size of most biosynthesised AgNPs cannot be precisely regulated, it has an impact on physiochemical properties of nanoparticles. For shape‐controlled AgNPs production, some studies employed compounds such as cetyltrimethylammonium bromide, amphiphilic compounds, detergents, anionic, and cationic agents [45]. But no empirical knowledge or reason was given to illustrate how the factors or phytoconstituents influenced the shape of green synthesised AgNPs, and no scientific proof or argument was supplied to show how the phytochemicals influenced the shape of green synthesised AgNPs.

## 6 CHARACTERISATION

Nanoparticles' physicochemical characteristics are essential for their actions, biodistribution, safety, and effectiveness. As a result, the characterisation of AgNPs is vital to assess the functional properties of the synthesised particles by various analytical techniques, including UV‐visible spectroscopy, X‐ray diffractometry (XRD), Fourier‐transform infrared spectroscopy (FTIR), dynamic light scattering (DLS), X‐ray photoelectron spectroscopy (XPS), transmission electron microscopy (TEM), scanning electron microscopy (SEM), energy‐dispersive X‐ray spectroscopy (EDX), selected area electron diffraction (SAED), and atomic force microscopy (AFM). Multiple reviews had already displayed the principles and application of various types of analytical methods; however, the basics of the essential method used for the characterisation of AgNPs have been described in detail below for easy comprehension.

### 6.1 UV‐visible spectroscopy

**FIGURE 5:**
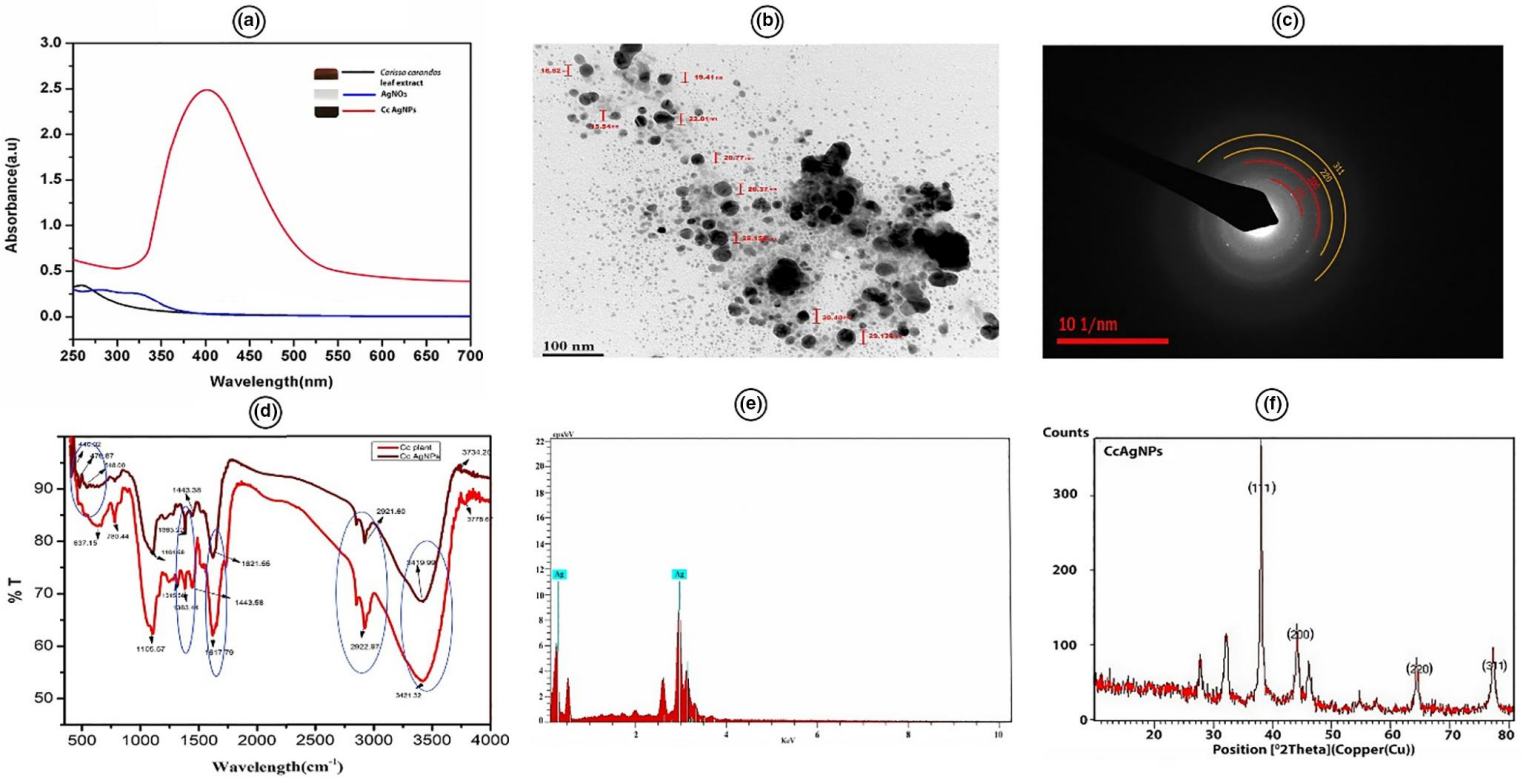
Different characterisation techniques of silver nanoparticles (AgNPs). (a) UV‐vis spectra of plant‐mediated synthesised AgNPs, silver nitrate (AgNO3), and plant extract. (b) Transmission electron microscopy (TEM) image of plant‐mediated synthesised AgNPs. (c) Selected area electron diffraction (SAED) pattern of plant‐mediated synthesised AgNPs. (d) FTIR spectra of plant‐mediated synthesised AgNPs and plant extract. (e) EDX spectra of plant‐mediated synthesised AgNPs. (f) X‐ray diffractometry (XRD) peaks of plant‐mediated synthesised AgNPs

UV‐vis spectroscopy is indeed a very valid and effective method for the primary characterisation of synthesised nanoparticles as well as monitoring the synthesis and stability of AgNPs. AgNPs have special properties that allow interaction strongly with specific wavelengths of light. Furthermore, UV‐vis spectroscopy is quick, simple, convenient, delicate, specific for different kinds of NPs, necessitates only a short measurement time, and no calibration is needed for the nanoparticle characterisation [46]. Absorption is affected by particle size, dielectric medium, and chemical environment. In AgNPs, the conduction and valence bands are relatively close to one another, enabling electrons to travel easily. These free electrons give rise to the SPR absorption band due to the collective oscillation of electrons of metallic nanoparticles in resonance with the light wave. The production of AgNPs was monitored by UV‐vis spectra, with the maximum reduction and formation of metallic silver nanoparticles determined by the absorbance intensity [47]. Figure 5a shows the UV‐vis spectra of the synthesised AgNPs, AgNO_3_, and plant extract. The intense peak at 410 nm by UV‐visible absorption spectra confirmed the formation of colloidal AgNPs [38]. The consistency of AgNPs prepared using biological methods has been evidenced for more than a year, and an SPR peak at the same wavelength has been witnessed using UV‐vis spectroscopy. However, UV‐vis spectroscopy alone would be insufficient for supplying detailed information about AgNPs.

### 6.2 X‐ray diffractometry

XRD is a widely used analytical method for analysing molecular and crystal structures, a qualitative identifier of active compounds, qualitative resolution of different molecules, able to measure crystallinity, isomorphous substitution, and particle size [48]. When X‐ray light is reflected on any particles, it creates a plethora of diffraction peaks, which represent the physicochemical properties of the crystalline lattice [49]. XRD could be used to examine the structural characteristics of a wide variety of materials, including inorganic catalysts, superconductors, biomolecules, glasses, and polymers. The configuration of diffraction peaks is crucial to the assessment of these materials' properties. Each material has a distinct diffraction light source that could be used to identify the content by correlating the diffracted beams to the Joint Committee on Powder Diffraction Standards (JCPDS) reference database. Bragg's law is the working concept of XRD. The size of AgNPs was determined using Debye‐Scherrer formula.

D=Kλ/β1/2cosθ

*D* = average crystalline size, *β* = line broadening in radians, *λ* = X‐ray wavelength, *Ɵ* = Bragg's angle, *K* = constant (geometric factor = 0.94). Figure 5f shows the XRD peaks of plant‐mediated synthesised AgNPs. The characteristic peaks at 38.01, 44.13, 64.46, and 77.40; Bragg's reflection corresponding to [110], [199], [220], and [311] lattice plans of the face‐centred‐cubic (FCC) structure of AgNPs were observed. This pattern shows the crystalline structure of AgNPs and size was estimated to be 25.4 nm by using Scherrer formula [38].

### 6.3 Fourier‐transform infrared spectroscopy

The biomolecules linked to the silver and surface atoms of the capping agents of the nanoparticles have been mostly analysed using FTIR spectroscopy. FTIR can give precision, repeatability, a good transmission ratio, and determine whether biomolecules are actively connected to the development of nanoparticles. Additionally, FTIR has indeed been applied to the investigation of nanoscaled materials, including the validation of functional molecules covalently bonded to silver, carbon nanotubes, graphene, and gold nanoparticles, or enzyme‐substrate connections during the catalytic reaction. Functional groups play a major role in capping as documented in the previous studies [50, 51]. The occurrence of peaks shows that the secondary metabolites of plants such as flavonoids, terpenoids, phenols, glycosides, and tannin role groups such as aldehydes, ketones, carboxylic acid were coated with the NPs. The elevated amounts of antioxidants and flavonoids (ascorbic acid and gallic acid) are responsible for the reduction of metal salts in crude extracts [52]. Figure 5d shows the FTIR spectra of plant‐mediated synthesis of AgNPs and plant extracts. The absorption band is due to the vibration effect of phytochemicals present in the plant extract, which is responsible for capping and stabilisation of AgNPs [38]. FTIR could be able to confirm that the amino acid residue and protein carbonyl group have a stronger ability to bind the metal and might prevent agglomeration.

### 6.4 Dynamic light scattering

DLS can investigate the size and dispersion of tiny particles on a scale that ranges from submicron to 1 nm. DLS is a technique based on light's interaction with nanoparticles. This technique could be used to quantify narrow size distribution, particularly in the 2–500 nm range. It uses Rayleigh scattering to quantify the scattered light from a beam passing through the particles [53]. The hydrodynamic particle size and size dispersion could be measured by analysing the variation of light scattering intensity as a proportion of time [54]. The characterisation of any nanomaterial in a solution is required to assess its hazardous capability. The size acquired by DLS is frequently bigger than that obtained by TEM, which could be attributed to Brownian motion [55]. The average diameter of nanoparticles dispersed in liquids may be determined using DLS. It gives the benefits of rapidly scanning a high number of particles, yet this has a variety of sample restrictions.

### 6.5 X‐ray photoelectron spectroscopy

XPS is an exceptional technique in that it opens a door to qualitative, quantitative/semi‐quantitative, and speciation data on the surface. XPS is carried out in a high vacuum environment and X‐ray irradiation of the nanoparticles induces electron emission; XPS peaks are obtained by measuring the kinetic energy and the number of electrons exiting from the surface of the nanoparticles. Kinetic energy could be used to determine the binding energy [56, 57].

### 6.6 Transmission electron microscopy with selected area electron diffraction

TEM is a significant, widely employed, and vital technology for obtaining the numerical value of particle size, particle distribution, and shape [57]. The distance between the objective lens and the sample as well as the distance between the objective lens and its picture plane defines the magnification of TEM [58]. TEM has two important benefits over SEM; it has higher resolution and could do more analytical investigations. The downsides are a high vacuum requirement, tiny sample portion, and a necessary component of TEM which is that sample preparation takes a lot of time. Another useful imaging technique for investigating the crystalline nature of nanoparticles is SAED. Electron backscatter diffraction investigations are commonly carried out in TEM where the electrons are accelerated by an electrostatic attraction to achieve the desired velocity and frequency prior to interacting with the sample that is to be analysed. Figure 5b shows the TEM microscopic image of plant‐mediated synthesised AgNPs. AgNPs was polydispersed and predominantly found to be spherical with the average diameter of approximately 14 nm [38]. SAED could decide the scale, shape, and distribution of particles and the crystalline nature of the particles [57]. Figure 5c shows the SAED pattern image of plant‐mediated synthesised AgNPs, which revealed the FCC of silver [38]. Because of the comparatively large size of the lightened area, the SAED approach is hampered by the feature that numerous nanoparticles interact with the diffraction peaks, leaving an individual investigation challenging.

### 6.7 Scanning electron microscopy with energy‐dispersive X‐ray spectroscopy

SEM is a surface imaging technique that can resolve various particle sizes, particle dispersion, nanoparticles architectures, and the surface morphology of produced nanoparticles at micro and nanoscales [59]. By manually quantifying and calculating the particles or by utilising the appropriate software, we could investigate the morphology of nanoparticles and generate a histogram from the SEM images. To evaluate the silver nanoparticle morphology and to perform elemental analysis, a combo of SEM and EDX could be employed. Figure 5e shows the EDX spectra of plant‐mediated synthesised AgNPs that confirms the presence of the silver element [38]. SEM has a major drawback of not being able to discern interior structures, but it could offer useful data about nanoparticle quality and level of agglomeration.

### 6.8 Atomic force microscopy

AFM has been employed to study nanoparticle dispersion, aggregation, size, shape, and structure. AFM could be used to characterise the interaction of nanoparticles with supported lipid bilayers in real time, which is not possible in the existing electron microscopy techniques [60]. AFM does not need an oxide‐free conductive surface for assessment. However, because of the size of the crossbar, there is an exaggeration of the lateral dimensions of the samples. As a response, we must pay close attention to prevent generating incorrect measurements.

## 7 BIOMEDICAL APPLICATION

A potential outbreak of antibiotic‐resistant microbes in a global scale demands the introduction of novel bactericidal agents to treat the infection and to create a platform to know more potent anti‐microbial agents that encounter multidrug resistance (MDR) pathogens [61]. AgNPs are well‐known for their efficient biocidal impact on microorganisms that have been employed for decades to restrain and treat various infections [62]. AgNPs have been widely used in domestic items, canned goods, medical sector, environmental, and biological purposes due to its various feature. The present study addresses various biological features of AgNPs, with an emphasis on antibacterial, antibiofilm, antifungal, antiviral, antidiabetic, antiulcer, blood compatibility, antiparasitic, antioxidant, anticancer properties, AgNPs for dental applications, drug delivery, wound healing activity, and several other applications were also discussed.

### 7.1 Antibacterial activity

**FIGURE 6:**
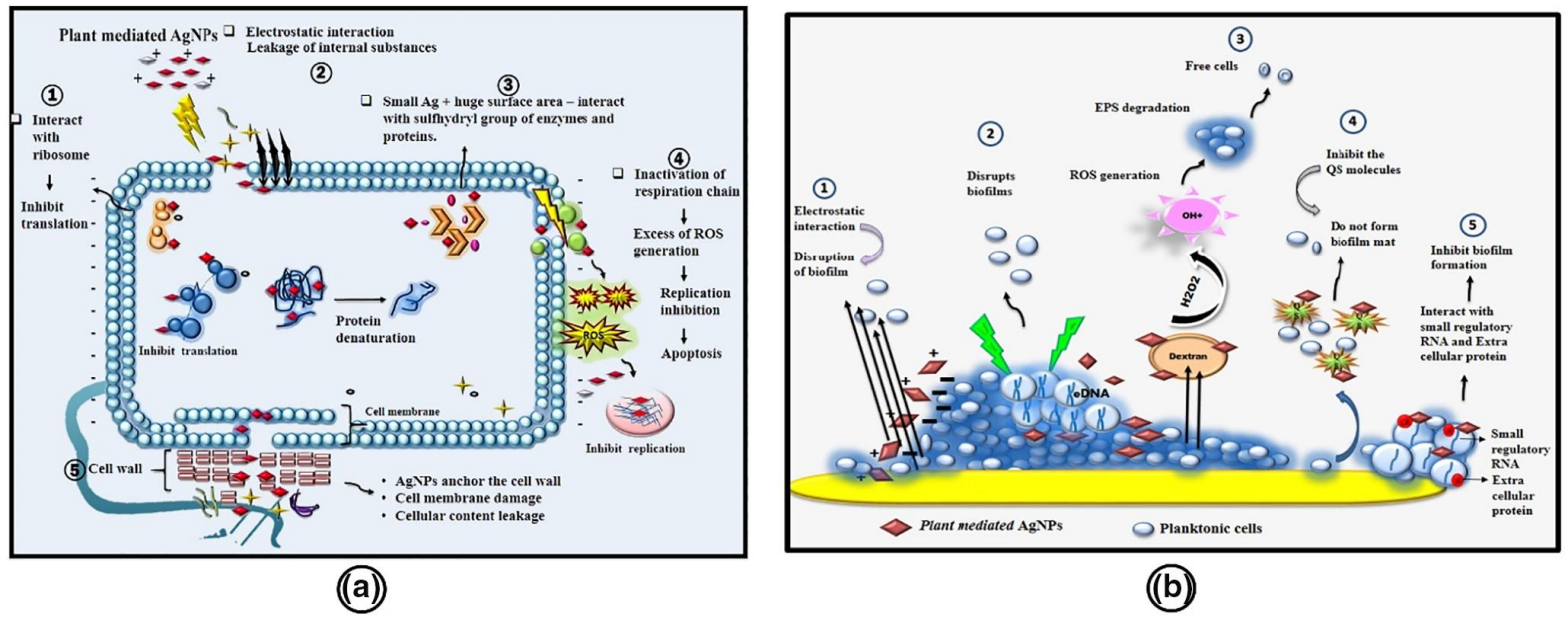
(a) Antibacterial mechanism of plant‐mediated synthesised silver nanoparticles (AgNPs) [38] (b) Antibiofilm mechanism of plant‐mediated synthesised AgNPs [38]

AgNPs are promising candidates for the creation of unique and effective biocompatible nanoscale components for novel antibacterial applications. AgNPs have a destructive action on the bacterial cell wall by inhibiting the cell respiration chain and cell nucleic acid (DNA or RNA) [63]. AgNPs have been developed as antimicrobial agents against MDR bacteria due to their large surface‐area‐to‐volume ratio and specific chemical and physical properties. The surface‐area‐to‐volume ratio is high as the particle size decreases [64]. AgNPs showed potential antibacterial activity due to their stability and size of the particle [65]. The size and shape of NPs are related to their antimicrobial property. AgNPs, which are smaller in size, have a more excellent binding surface when compared with larger AgNPs and show a greater bactericidal activity [66, 67]. The plasma membrane is attracted to the positively charged silver ions when the charges of these particles (AgNPs) come together, major conformation shifts take place in the membrane, thereby gradually losing its permeability, leading to cell death [68]. AgNPs communicate with the biomaterials rich in sulphur and phosphorus, including cellular constituents that significantly impact respiration, cell division, and consequently cell survival [69]. Furthermore, particle size and shape play a significant role in the antimicrobial properties of NPs. NPs with small angles have better antimicrobial properties when compared with spherical and rod‐shaped particles [70]. Due to microbial resistance to antibiotics caused by mutation, discoveries of antibacterial products or agents are desperately needed. AgNPs were also ideal for clinical and therapeutic use due to their reduced reactivity compared to silver ions. AgNPs have four known antibacterial properties: (1) adhesion to microorganism cell membranes, which causes damage; (2) penetration of AgNPs into cells, which disrupts biomolecules and intracellular damage; (3) induce cellular toxicity by generating reactive oxygen species (ROS), which causes oxidative stress in cells; and (4) disruption of cell signal transduction pathways, which causes oxidative stress. There are several AgNPs antibacterial mechanisms available in the literature. We have proposed the antibacterial mechanism of plant‐mediated synthesised AgNPs in our previously published article. Figure 6a illustrates the antibacterial mechanism of AgNPs synthesis using plants [38]. (1) AgNPs link with the ribosomes and restrict translation; (2) AgNPs possess electrostatic interactions with the outer membrane, resulting in the internal material spillage; (3) AgNPs bind with the sulphydryl class of enzymes and proteins, causing protein aggregation; (4) AgNPs directly inhibit the respiratory chain resulting in death; (5) AgNPs attaches the bacterial cell wall, causing disruption of the cell membrane and leakage of cellular content [38].

Table 1 shows some of the plant‐mediated synthesised AgNPs’ antibacterial activity with their morphological characterisation. Few examples of the synthesis of AgNPs from medicinal plants exhibiting antibacterial activity are as follows: AgNPs mediated by *Acorus calamus* (rhizome) extract showed a more excellent activity against *Staphylococci aureus*, *Salmonella enterica*, *B. cereus,* and *S. enterica, E. coli* in a dose‐dependent manner as well as exhibiting good stability and could be used as a potential antibacterial agent for a commercial application [73]. AgNPs synthesised using aqueous *Allium sativum* extract counteracts with the test bacterial strains *Staphylococcus aureus* and *Pseudomonas aeruginosa* [74]. *Acalypha indica* aqueous extract‐mediated AgNPs showed activity against *P. aeruginosa* (16 mm), *Escherichia coli* (14 mm), *S. aureus* (13 mm) and *Bacillus subtilis* (12 mm). The biosynthesis of AgNPs by flavonoids present in the petals of *Linium casa blanca* exhibited good stability, antibacterial and catalytic activity. It showed greater antibacterial activity against *Escherichia coli* and *Salmonella*, lesser antibacterial activity of AgNPs was found against *Bacillus subtilis* and *Staphylococcus aureus* [102]. The green‐mediated synthesis of AgNPs using *Myristica fragrans* dried seed extract showed good bactericidal activity against MDR *Salmonella enterica* bacteria (ciprofloxacin, tetracycline, and cefotaxime resistant) and the rising concentration of AgNPs with superior stability increases the activity of AgNPs [86]. AgNPs using *Boerhaavia diffusa* and assessed against fish pathogens, namely *Pseudomonas fluorescens, Aeromonas hydrophila, and Flavobacterium branchiophylum*. AgNPs showed high potential antibacterial activity in *F. branchiophylum* bacteria than other test bacterial pathogens [30]. Formulation of AgNPs mediated by *Skimmia laureola* is effective against human pathogens *E. coli* (11.67 mm), *Pseudomonas aeruginosa* (14.33 mm), and *Staphylococcus aureus* (14.67 mm). AgNPs were spherical and hexagonal in shape and crystalline where silver is reduced and stabilised by capping with the phytochemicals present in the *Skimmia laureola* extract [103]. AgNPs of *Aloe vera* (leaf) origin has potential antibacterial activity. The flavanoids and terepenoids present in the extract were responsible for the stabilisation of the AgNPs with the octahedran shape exhibiting higher antibacterial properties against *Bacillus cereus, S. aureus, Micrococcus luteus, E. coli, and K. pneumoniae* [75]. AgNPs synthesised using the aqueous extract of *Lawsonia inermis* exhibited antibacterial properties against gram positive alpha and beta haemolytic *Streptococcus sps., Streptococcus aureus, S. haemolyticus, and Bacillus sps.,* and gram‐negative bacteria such as *E. coli, E. faecalis, Proteus mirabilis, Klebsiella pneumonia, and P. aeruginosa*.

**TABLE 1:** Antibacterial action of various medicinal plants and its parts mediated synthesised silver nanoparticles (AgNPs) with their morphological distribution

S.No	Plant name (part)	UV‐vis SPR (nm)	Size (nm)	Morphology	Possible biomolecules involved in the reduction and stabilisation	Applications	Reference
1	*Acacia nilotica* (leaf)	300	10–50 (SEM)	Spherical, uniform distribution	Proteins and phenolic compounds arising from carbonyl and OH stretching	Antibacterial activity	[71]
2	*Acalypha Indica* (entire plant)	390–410	0.516 (particle size analyser)	Spherical	Polyphenols, and alkaloids	Antibacterial (*B. subtilis, S. aureus, P. aeruginosa, E. coli*) activity	[72]
3	*Acorus calamus* (rhizome)	420	20–35 (SEM)	Spherical	Amino, carboxylic, hydroxyl, carbonyl groups predominantly capping of amide containing compounds	Antibacterial against human pathogens *S. aureus* and *E. coli*	[73]
4	*Allium sativum* (cloves)	415	7.3 ± 4.4	Poly‐dispersed spherical	Flavanones, and terpenoids	Antibacterial potential against *S. aureus, P. aeruginosa*	[74]
5	*Aloe vera* (leaf)	420	5–50 (FE‐SEM)	Octahedron	Flavanones, and terpenoids	Antibacterial (*Staphylococcu s aureus, Bacillus cereus, E. coli, Klebsiella pneumoniae, Micrococcus luteus*) activity	[75]
6	*Aristolochia bracteata* (leaf)	441–423	12 (TEM)	Spherical	Proteins, alcohol, phenol, and amine groups	Antibacterial activity (*E. coli, B. subtilis*)	[76]
7	*Artocarpus heterophyllus* (seed)	410–420	10.78 (TEM)	Irregular shape	Amides, carboxyl, amino groups and amino acid residues	Antibacterial (*B. cereus, B. subtilis, S. aureus, P. aeruginosa)* activity	[77]
8	*Azadirachta indica* (leaf)	436–446	34 (DLS)	Irregular shape	Amide, alkyne functional groups and flavonoids, terpenoids excessively present	Antibacterial (*S. aureus, E. coli*) activity	[19]
9	*Boerhaavia diffusa* (entire plant)	418	25 (XRD)	Spherical	O‐H bond and NO_3_^−^	Antibacterial activity against fish pathogens (*A. hydrophila, P. fluorescens and F. branchiophilum*)	[30]
10	*Elettaria cardamomum* (seed)	440–480	40–70 (SEM)	Spherical	Alcohols, carboxylic acid, ethers, esters, and aliphatic amines.	Antibacterial activity (*Bacillus subtilis, Klebsiella planticola*)	[78]
11	*Cassia angustifolia* (leaf)	420	21.6 (TEM)	Spherical	Sennosides	Antibacterial potential (*E. coli, S. aureus*)	[79]
12	*Chrysanthemum indicum* (flowers)	435	37.71–71.99 (SEM)	Spherical	Flavonoids, terpenoids, and glycosides	Antibacterial effect against (*Klebsiella pneumonia, Escherichia coli, and Pseudomonas aeruginosa)*	[80]
13	*Commiphora wightii* (leaves)	200–600	‐	‐	‐	Antibacterial activity against *E. coli*, *P. aeruginosa, B. subtilis* and *S. aureus*	[81]
14	*Embelia Officinalis* (fruit)	432–436	10–70 (AFM)	Spherical	Tannins, alkaloids, phenolic compounds, amino acids, carbohydrates	Antibacterial activity (gram positive *S. aureus* and *B. subtilis,* gram negative *E. coli* and *K. pneumonia*)	[82]
15	*Gymnema sylvestre* (leaf)	442	20–30 (TEM)	Spherical	Amines, aliphatic amines, carboxylic acid, alcohol	Antibacterial activity *S. aureus* and *E. coli*	[83]
16	*Lantana camara* (leaf)	421	37–29 (FE‐SEM)	Spherical	Flavonoids, terpenoids, alkaloids	Antibacterial activity against *Bacillus spp., Pseudomonas spp.*	[84]
17	*Tagets erecta* (flower)	430	10–90 (TEM)	Spherical, hexagonal and irregular	‐	Antibacterial activity against (*S. aureus, B. cereus, E. coli and P. aeruginosa*)	[85]
18	*Myristica fragrans* (seed)	420	25 (TEM)	Spherical	Carbonyl, alkene, amine	Antibacterial activity against MDR *Salmonella enterica* serovar Typhi isolates	[86]
19	*Nigella sativa* (seed)	450–480	1–100 (SEM)	Irregular	‐	Antibacterial activity against *Staphylococcus aureus and Escherichia coli*	[87]
20	*Ocimum sanctum* (leaves)	430	0–50 (SEM)	Spherical	Alkaloid, and flavonoids	Antibacterial against ‐*Staphylococcus aureus, Staphylococcus saprophyticus, Escherichia coli, Klebsiella pneumoniae, Enterococcus faecalis, Enterobacter cloacae, and Proteus vulgaris*	[88]
21	*Olive (leaf)*	440–458	20–25	Spherical	Oleuropein, apigenin‐7‐glucoside or luteolinn‐7‐glucoside	Antibacterial activity against MDR *S. aureus, P. aeruginosa and E. coli*	[89]
22	*Phoenix dactylifera* (root hair)	420	15–40	Spherical	Alkenes, alcohols, carboxylic acid, esters and ethers	Antibacterial activity against *E. coli, C. albicans*	[90]
23	*Phyllanthus amarus* (leaf)	404	‐	‐	Hydroxyl, and carbonyl groups	Antibacterial activity – *B. subtilis, E. coli*	[91]
24	*Crocus sativus* (Wastages)	450	15 (TEM)	Spherical	‐	Antibacterial activity against *E. coli, P. aeruginosa, K. pneumonia, Shigella flexneri and B. subtilis*	[52]
25	*Saraca asoca* (leaves)	‐	24.85 (AFM)	Granular	Secondary metabolites (flavonoids, alkaloids, proteins and polyphenols)	Antibacterial activity against *Staphylococci aures, Streptococci pyogens, Salmonella typhi*	[92]
26	*Sesamum indicum* (seed)	430	‐	‐	Carbohydrates, and proteins	Antibacterial activity against multi drug resistant *E. coli*	[93]
27	*Sesbania grandiflora* (leaf)	422	10–25 (AFM)	Spherical	Amide I band, proteins having functional groups of amines, alcohols, ketones and carboxylic acids	Antibacterial activity against multi‐drug resistant *Salmonella enterica* and *Staphylococcus aureus*	[60]
28	*Skimmia laureola* (leaves)	460	40 (SEM)	Irregular spherical, hexagonal	Skimmidiol	Antibacterial activity against human pathogens	[67]
29	*Skimmia laureola* (fruit)	430	5–50 (SEM)	Spherical	Flavonoids, terpenoids, and soluble proteins	Antibacterial activity against *E. coli, Pseudomonas* and *Bacillus*	[94]
30	*Solanum trilobatum* (fruit)	432	12.50–41.90 (SEM)	Spherical	Hydroxyl/amine	Antibacterial activity against *Streptococcus mutans, Enterococcus faecalis, Escherichia coli, Klebsiella pneumoniae*	[95]
31	*Solanum xanthocarpum* (berry)	406	10 (TEM)	Spherical	Steroidal alkaloids, carbohydrates, flavonoids, terpenoids, and proteins	Antibacterial and urease inhibitory activities against *Helicobacter pylori*	[96]
32	*Swertia paniculata* (aerial parts)	423–432	31–44 (HR‐TEM)	Spherical	Xanthones, and flavonoids	Antibacterial activity against *Pseudomonas aeruginosa,* and *Klebsiella pneumoniae*	[97]
33	*Tectona grandis* (leaf)	450–490	30–40 (TEM)	Spherical to oval shape	Proteins and terpenoids	Antibacterial activity against *Staphylococcus aureus, E. coli*	[98]
34	*Urtica dioica* (leaves)	414	20–30 (TEM)	Spherical	Alcohol and phenols	Antibacterial activity against gram‐positive (*Bacillus cereus, Bacillus subtilis, Staphylococcus aureus* and *Staphylococcus epidermidis*) and gram‐negative (*Escherichia coli, Klebsiella pneumoniae, Serratia marcescens and Salmonella typhimurium*)	[99]
35	*Vitex Negundo* (leaves)	422, 447	18.2 ± 8.9 (TEM)	Spherical	Total phenolic compounds, and flavonoids	Antibacterial activity against *S. aureus and E. coli*	[100]
36	*Withania somnifera* (leaves)	430–450	12–36 (SEM)	Spherical	Polyphenols, alkaloids, and flavonoids	Antibacterial activity against *Klebsiella pneumoniae, Staphylococcus aureus*	[101]

After establishing a stable bonding with the plant extract, AgNPs enter the bacteria and rupture the cell membrane, causing cell death and also act as an oxidizing agent on the surface of the plasma membrane. AgNPs are known for their significantly higher antimicrobial property and the medicinal plant‐mediated AgNPs showed greater activity than the plant extract. Moreover, the antibacterial property of AgNPs depends on the stability of the particle and this plays a vital role in suppressing microorganism proliferation and development. In addition to that, AgNPs along with commercial antibiotics showed a greater synergistic effect. From these studies, we can assume that biogenic AgNPs have the potential to be a valuable biomaterial for biomedical applications.

### 7.2 Anti‐biofilm activity

The antibiofilm action of AgNPs has only been studied in a restricted manner. The production and secretion of EPS is responsible for the formation of biofilm [104]. Table 2 shows antibiofilm activity of various plant‐mediated synthesised AgNPs with their morphological characterisation. The effectiveness of the root extract of the *Vetiveria zizanioides* mediates the synthesis of AgNPs (VzAgNPs) and it showed potent anti‐QS and anti‐biofilm activity against *Serratia marcescens.* AgNPs have been discussed to attenuate the virulence factor of biofilm formation, which is QS‐dependent, namely prodigiosin, protease, lipase, and the development of extracellular polymeric substances. Transcriptomic research validates the regulation of quorum‐sensing genes that are encoded to develop virulence factors. VzAgNPs have greater stability resulting in the enhanced antibiofilm activity [106]. AgNPs synthesised using *Withania somnifera* (L) showed a broad spectrum inhibitory effect on microbial growth by disrupting the cell membrane leading to the leakage of cellular content [107]. *Artemisia vulgaris* extract‐mediated AgNPs has biofilm reduction capacity and anthelmintic activity. This activity revealed the paralytic effect and could be a potential alternative to treat biofilm formed by multidrug‐resistant pathogens. *A. vulgaris* rich in flavonoids provides stability to the AgNPs, which impact the activity of silver nanoparticles [108]. *Piper beetle* (leaf) aqueous extract‐mediated synthesis of AgNPs shows anti‐quorum sensing and antibiofilm activity against the common urinary tract infection pathogens and assessed the anti‐quorum sensing activity of AgNPs by the inhibition of quorum‐sensed intermediated virulence factors (i.e.) protease (44%), prodigiosin (62%–72%), hydrophobic development in uropathogens and extracellular polymeric substance (EPS) formation. AgNPs can be utilised as a possible alternative to typical antibiotics to control the quorum‐sensing activity of the uropathogens associated with a biofilm. Gene expression downregulated the *fimA, fimc, flhD,* and *bsmB* genes in *S. marcescens* and *flhB, flhD,* and *rsbA* genes in *P. mirabilis,* which is an evidence for the biofilm inhibition [105, 109].

**TABLE 2:** Anti‐biofilm activity of various medicinal plants and its parts mediated synthesised silver nanoparticles (AgNPs) with their morphological distribution

S. No	Plant name (part)	UV‐vis SPR (nm)	Size (nm)	Morphology	Possible biomolecules involved in the reduction and stabilisation	Application‐Antiquorum sensing activity	Reference
1	*Piper betle* (leaves)	440	156.4 (DLS)	Spherical	Aliphatic amine	Antiquorum sensing and anti‐biofilm activity against *S. marcescens and P. mirabilis*	[105]
2	*Vetiveria zizanioides* (root)	445	20–60 (TEM)	Spherical	Aromatic amide, amide linkage of proteins	Antiquorum sensing and anti‐biofilm activity against *Serratia marcescens*	[106]

Biofilm‐forming bacteria have become resistant to antimicrobial agents due to the following factors: (1) the antibiotic's failure to enter the biofilm, (2) formation of multi‐drug resistance bacteria, (3) deactivation of drugs by antimicrobial enzymes or transformed induced by the biofilm. Plant‐mediated AgNPs could be an effective agent against antibiofilm formed by MDR pathogens and could find that AgNPs targets the biofilm formation and EPS against the pathogens, which makes the silver nanoparticles efficient against the microbes. The antibiofilm mechanism has been proposed in our previous work as shown in Figure 6b. (1) AgNPs connect electrostatically with cells, disrupting biofilm development; (2) AgNPs attack eDNA to destroy bacterial biofilm; (3) AgNPs impair EPS production, breaking the biofilm mat; (4) AgNPs suppress the bacteria's signal preventing the biofilm formation; (5) AgNPs engage with tiny regulatory RNA and extracellular protein to prevent biofilm formation [38].

### 7.3 Antifungal activity

Fungal infections are more common in immunocompromised people, and treating the illnesses associated with fungi is a time‐consuming procedure due to the minimal choice of antifungal medications that are presently accessible. As a consequence, there seems to be an inherent and immediate necessity to produce antifungal medicines that are biocompatible, non‐toxic, and ecologically benign. At this point, AgNPs play a significant role in antifungal medicines against a variety of fungi‐caused infections.

Table 3 shows antifungal activity of various plant‐mediated synthesised AgNPs with their morphological characterisation. *A. indica*‐mediated synthesised AgNPs showed potential antifungal activity against *A. niger* (12 mm) and *C. albicans* (23 mm) at 300 µg/ml concentration. AgNPs shows hopeful activity against fungal pathogen compared to the standard commercial antibiotic ketoconazole [72]. AgNPs were produced using *Lawsonia inermis* (Henna). The synthesis of phytochemical‐capped nanoparticles and its potency were studied against human pathogens such as *Microsporum canis*, *Trichophyton mentagrophytes,* and *Candida albicans. Microsporum canis, Trichophyton mentagrophytes*, and *Candida albicans*, which showed synergistic activity with commercial antibiotics. AgNPs exhibited greater stability and a greater synergistic effect with commercial antibiotics. The nanogels formulated using AgNPs also showed effective results against the pathogenic fungus and suggested in treating the skin infections by gel formulations or cream [111]. The anti‐fungal activity against *Aspergillus sp.,* using *Aloe vera* (leaves)‐mediated AgNPs showed higher activity. The maximum fungal hyphae inhibition was obtained at 10 μL of AgNPs and the microscopic results showed the destructive causes of the fungal conidial germination and structural deformation especially in the cell structure and budding process [113].

**TABLE 3:** Antifungal activity of various medicinal plants and its parts mediated synthesised silver nanoparticles (AgNPs) with their morphological distribution

S. No	Plant name (part)	UV‐vis SPR (nm)	Size (nm)	Morphology	Possible biomolecules involved in the reduction and stabilisation	Applications	Reference
1	*Acalypha Indica* (entire plant)	390–410	0.516 (particle size analyser)	Spherical	Polyphenols, and alkaloids	Antifungal (*C. albicans and A. niger*)	[72]
2	*Aloe vera* (leaf)	400	70 (SEM)	Cubical, rectangular, triangular and spherical	Primary amine, phenol, alcohol, and nitriles	Antifungal activity against *Rhizopus sp., Aspergillus sp*.	[75]
3	*Elettaria cardamomum* (leaves)	434	29.96 (TEM)	Irregularly spherical	Alkenes, amides, and alkyl halides	Antifungal activity (*Alternaria alternata, Aspergillus niger, Botrytis cinerea, Fusarium Oxysporum* and *Penicillium expansum)*	[110]
4	*Lawsonia inermis* (leaves)	445	5–45 (TEM)	Spherical	Aliphatic and aromatic amines	Antifungal nanogel against *Candida albicans, Microsporum canis, Propioniabacterium acne and Trichophyton mentagrophytes*	[111]
5	*Tagets erecta* (flower)	430	10–90 (TEM)	Spherical, hexagonal and irregular	Alkaloids, flavonoids, tannins, and cardiac glycoside	Antifungal activity against (*Candida glabrata, Candida albicans, Cryptococcus neoformans)*	[85]
6	*Ocimum sanctum* (leaves)	430	0–50 (SEM)	Spherical	Proteins, terpenoids having functional group of amines, alcohols, ketones, aldehydes, and carboxylic acids	Antifungal activity against *C. albicans, C. tropicalis, C. krusei, C. kefyr, A. niger, A. flavus, A. fumigatus*	[88]
7	*Phoenix dactylifera* (date palm)	428	27 (DLS)	Spherical	Aliphatic amines, carboxyl, hydroxyl, amine	Antifungal against *Rhizoctonia solani*	[112]
8	*Withania somnifera* (leaves)	430–450	12–36 (SEM)	Spherical	‐	Antifungal activity against *Candida albicans.*	[101]
9	*Phoenix dactylifera* (root hair)	420	15–40	Spherical	Alkenes, alcohols, carboxylic acid, esters, ethers.	Antifungal activity against *C. albicans*	[90]

**FIGURE 7:**
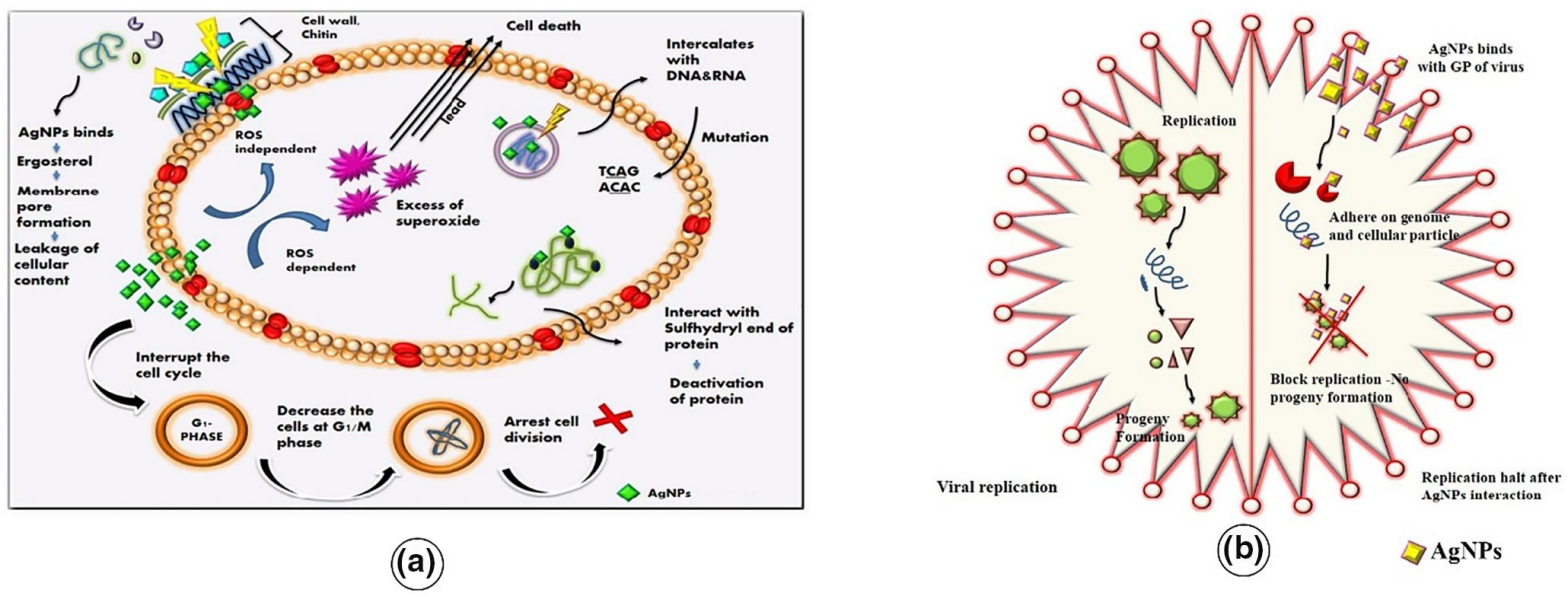
(a) Antifungal mechanism of plant‐mediated synthesised silver nanoparticles (AgNPs) [115] (b) An Illustrated antiviral mechanism of plant‐mediated synthesised AgNPs

AgNPs not only inhibit the human pathogens but also several indoor fungal pathogens, including *Aspergillus fumigatus, Cladosporium cladosporoides, Chaetomium globosum, Stachybotrys chartarum, Penicillium brevicompactum, Mortierella alpine,* and *Fusarium sp.,* [114, 115]. The precise mechanism of AgNPs' antifungal action is currently unknown. The antifungal mechanism of AgNPs was postulated and clarified in our previous work as shown in Figure 7a. [115] (1) AgNPs engage with the ergosterol in the cell wall to form a pore allowing the internal organelles to flow out; (2) AgNPs activate reactive oxygen species, and excessive production leads to apoptosis; (3) AgNPs connect with DNA and RNA, inhibiting cell division; (4) AgNPs attack the sulfhydryl end of protein, thereby preventing protein synthesis; and (5) AgNPs interfere with the G1/M phase, arresting cell division [115].

### 7.4 Anti‐viral property

Millions have died throughout the world, whereas others have lost their families, people are unemployed and youngsters have ended up losing access to good education, all of which has resulted in global economic problems [116]. The phyto‐compounds are excellent and promising options for the synthesis of green silver nanoparticles (AgNPs), which show significant promise in the treatment of chronic illness. The viral illness that would be the source of today's outbreak has spread fear throughout humankind and is destroying the globe. Some of the medicinal plants like *Andrographis paniculata* [117], *Olea eurolaea* [118], *Pachyma hoelen* [119], *Phyllanthus niruri* [120], *Tihenospora cordifolia* [121], and *Swetia chirata* [122] have shown virustatic properties against the viruses that cause diseases such as dengue, herpes, HIV. Other viruses, in addition to the corona virus, grow and multiply rapidly, causing life‐threatening illnesses such as HIV, influenza virus, nipah virus, ebola virus, and herpes virus [123].

Vaccines against the viruses are being developed by all pharmaceutical firms and researchers. Unfortunately, the rest of the globe is still recovering from it. This calls for immediate research and development of a novel antiviral medication to treat humans with life‐threatening infections [124]. AgNPs are believed to be an effective and potent pharmacological agent with antiviral action. Biologically synthesised AgNPs from *Phyllanthus niruri, Andrographis paniculata, and Tinosporacordi folia* were tested against CHIKV and *A. paniculata* AgNPs showed promising antiviral property [125]. These findings suggest that utilising the plant‐mediated AgNPs as antiviral medicines is viable and that they could give an alternative therapy for viral illness for which no specialised antiviral vaccine is currently available. AgNPs have been shown to exhibit predominant action against a variety of life‐threatening viruses, making them ideal for viral infection treatment. Antiviral drugs are significant since viral‐mediated diseases are widespread and becoming increasingly prevalent around the world. Antiviral therapies rely heavily on understanding the mechanism of AgNPs' antiviral property. Figure 7b illustrated the antiviral mechanism mediated by the AgNPs synthesised using medicinal plants. AgNPs communicate with the viruses and bacteria differently depending on their size and form. Although AgNPs have the potential to reduce the virus survival, the actual antiviral mechanism is still unclear. The interaction of AgNPs with the virion surface and viral core is responsible for viral replication mechanism. AgNPs binds with the glycoprotein of the virus and then adheres to genome and cellular particles, thereby blocking the viral replication process [126].

### 7.5 Anti‐diabetic activity

Diabetes is a metabolic illness characterised by an increased glucose content in the blood that affects over 100 million individuals globally. Diabetes has become a serious concern to people's health because it includes the pancreas' failure to generate enough insulin for blood sugar control or the body cells' difficulty to utilise the insulin that is produced [127]. Inhibiting carbohydrate‐digesting enzymes (α‐glucosidase and α‐amylase) is one method of treating hyperglycemia by limiting the digestion of starch into monosaccharides, which is the biggest factor causing high blood glucose levels. Therefore, creating drugs that inhibit the carbohydrate digesting enzymes could be an effective strategy to treat diabetes. AgNPs formulated using *Tephrosia tinctoria* (stem) extract significantly showed carbohydrate digestive enzymes (alpha‐glucosidase and alpha‐amylase) inhibition, where glucosidase and amylase are important targets for preventing the sudden increase in glucose levels in the blood at high concentrations and increases the glucose uptake in the cells [128]. AgNPs scavenges the free radicals (3.8 ± 0.028) and the results showed higher glucose uptake compared to crude extract (2.61 ± 0.07) at 75 μg/ml concentration of AgNPs and increased glucose uptake at concentrations, enzyme inhibition was observed in an increased order TT < AgNPs < acarbose. This medicinal plant was rich in phenol and flavonoid groups of compounds. AgNPs were crystalline in nature and exhibited bioactivity and also it has the antioxidant activity with no haemolysis during the glucose uptake process [128]. The ability of AgNPs synthesis using *Pterocarpus marsupium* extract controls the blood sugar level, and the enzyme amylase was inhibited. The amylase inhibition percentage of *Pterocarpus marsupium*‐mediated AgNPs was 84.09%. The synthesised AgNPs was stable and showed a promising antidiabetic factor for a long period. The studies on plant‐mediated AgNPs showed significant anti‐diabetic activity against carbohydrate‐inhibiting digestive enzymes. Plant‐mediated AgNPs were discovered to have exceptional potential antidiabetic activity against major diabetes enzymes and is suitable for nanomedicine and nano biomedical properties.

### 7.6 Anti‐ulcer activity

*Helicobacter pylori*, one of the most common causes of peptic ulcers and cancer, has become increasingly resistant throughout the world. Therefore, novel antimicrobial drugs are needed to combat the negative impacts of antibiotic therapy, particularly in the case of *H. pylori*. Because of the unique physicochemical features of nanomaterials, nanotechnology is indeed a notable domain for researchers looking for medication possessing significant antibacterial capabilities. The development of various therapeutic candidates has rapidly fuelled research into the AgNPs for reducing bacterial infections. The AgNP synthesis mediated by *Solanum xanthocarpum* L (SXE) was reported by [96] Amin M et al., where the extract from berry was analysed to test against *H. Pylori* pathogen, which is the causative agent of gastric and duodenal ulcers. AgNPs was found to be monodispersed in nature, spherical in shape, and highly stable. SXE acted as both a reducing and capping agent. *Solanum xanthocarpum*‐mediated AgNPs was examined against 34 clinical isolates with *H. pylori* strain as a reference by in‐vitro technique. The action of AgNPs compared with silver nitrate (AgNO_3_) and standard drugs, that is amoxicillin (AMZ), metronidazole (MNZ), tetracycline (TET), and clarithromycin (CLA), has higher *H. pylori* activity. AgNPs product typically inhibits the growth of *H. pylori* activity than AgNO_3_, MNZ, TET, AMZ, and CLA, which exhibited urease inhibitory activity. In‐vitro anti‐ulcer activity of medicinal plant‐mediated AgNPs have an effect on *H. pylori* for the treatment of gastric ulcer and provides a promising approach for gastric ulcer therapy. AgNPs were found to be effective against both the antibiotic‐resistant and antibiotic‐susceptible strains of *H. pylori*. One of the really obvious antibacterial strategies would be that Ag+ ion produced from the AgNPs could disrupt cell growth by tightly attaching to thiol groups on the cell membrane, resulting in bacterial cell lysis. Furthermore, AgNPs could induce oxidative stress by releasing ROS, which might target the enzymes and proteins, causing permanent DNA damage.

### 7.7 Blood compatibility

Blood coagulation problem, which can lead to cardiovascular disease, is a major health concern in humans. Cardiovascular illnesses are a leading cause of death globally, accounting for more than 17.7 million deaths in 2015. The effect of AgNPs on different types of cells is found in the complicated vascular system, but the findings have been conflicting. The recorded information, on the other hand, can support the development of innovative and precise molecular treatments in vascular function, vasopermeability, and angiogenesis. Cardiovascular disease, including hypertension, may have an impact on AgNP‐induced toxicity. Nanotechnology has emerged as a tool for providing high‐quality healthcare. It has been reported that AgNPs were translocated to the blood circulation and distributed throughout the major organs like kidney, liver, spleen, and endothelial cells of the blood–brain barrier [129]. The biosynthesis of AgNPs by Ginger (*Zingiber offficinale*) extract provides greater blood compatibility, which is a serious issue when silver‐mediated products are served as a therapeutic agent. *Zingiber officinale‐*mediated AgNPs are extremely robust with respect to the physiological state and are compatible with blood and support as a vector for drug delivery. AgNPs synthesised using *Zingiber offficinale* was highly stable, which supports its usage as vectors for applications in drug delivery, gene delivery, or as biosensors that are directly in contact with blood [130]. Additionally, the extracts of *Zingiber offficinale* have high medicinal value and their pretreatment suppress induced hyperglycemia and hypoinsulinaemia and considered as a potential antiplatelet agent than aspirin [131]. Synthesised AgNPs have potent biological effect on blood and were unaffected by platelet aggregation, coagulation cascade, and complement system activation.

### 7.8 Silver nanoparticles for dental applications

Tooth decay is one of the most widespread oral cavity‐related diseases in the world as well as a substantial financial load. Nanotechnology‐based solutions for dental problems attempt to restrict or indeed remove the therapeutic significance by boosting restoration of minerals and suppressing biofilm colonisation [132]. Components for the dental membrane are biocompatible, which are usually employed for successful oral bone regeneration, also should fulfil several particular and extra properties and functions. Several metal‐coated implants were tested for resistance to microorganisms that develop biofilm and eventual implant failure [133]. The key purpose of dentistry is to safeguard the mouth cavity that serves as an entry point for harmful pathogens. Acute lesions in the implant mucosa could be caused by the biofilm formed on the implant placement surfaces [134]. AgNPs synthesised from *Mangifera indica* leaves have better stability with promising antimicrobial activity against *S. aureus* and *E. coli*. To strengthen the mechanical bonding of glass ionomer cement (GIC) AgNPs were integrated into GIC in 2% weight ratio. Vickers and Monsanto microhardness test ensures the hardness level of GIC and GIC along with nanosilver and micro silver particles. Promising outcomes were seen by GIC improved with nanosilver particles (14.2 Kg/cm^2^) compared to GIC with microparticles (9.5 Kg/cm^2^) and GIC alone (11.7 Kg/cm^2^). Vickers hardness results demonstrated that GIC property was improved when GIC integrated with AgNPs (82 VHN) when compared with GIC reinforced with microparticles (61 VHN), and GIC alone (54 VHN). This study suggests dual dentistry application of the synthesized AgNPs [135].

Silver has been applied in dental hygiene for millennia and acquired widespread recognition as primary material in dental amalgams. AgNPs were employed in a wide variety of dental applications, including artificial teeth, therapeutic, and endodontic dentistry [136]. Antibacterial resins have the potential to be employed in clinical dental treatments including orthodontics and regenerative dentistry. The technique for integrating AgNPs into acrylic resin dental composites were designed to boost their mechanical characteristics and antibacterial effects. AgNPs could be used as a bactericidal coatings for traditional titanium dental implants [137]. Despite the fact that AgNPs have shown to be effective and beneficial medicines in dentistry treatment, they remain a problematic option due to their diverse toxicities in biomolecules. As a result, any interesting approaches of AgNPs in dentistry should incorporate extensive research in physicochemical properties and biocompatability.

### 7.9 Anti‐parasitic activity

Despite the progress in the medicine field, mosquitoes in all tropical and semitropical regions are fully accountable for pathogen transmission, which can cause the most fatal and debilitating diseases, including yellow fever, filariasis, malaria, dengue, chikungunya, encephalitis, and so on. The prolonged use of artificially engineered vector control insecticides involves changing and disrupting the natural biological system. Hence, the requirement of bio‐control agents is crucially important [138]. Larvicides made from herbal ingredients are indeed an exciting and innovative type of pesticide due to their low toxicity for non‐target organisms and lesser ecological contamination. Traditional insecticides typically have one active ingredient, whereas the plant‐derived larvicides typically have a combination of numerous phytochemical compounds that interact together, targeting different biological processes thereby minimising the probability of tolerance in the target organism. This approach combines the silver's microbicide characteristics and the insecticidal activity of the chosen plant extract. The high efficacy could be achieved by the favourable nanoparticle size and shape. The biosynthesis of AgNPs mediated by *Morinda tinctoria* assesses the effectiveness of the AgNPs against third instar larvae *C. quinquefasciatus.* AgNPs was stabilised and reduced by the phytoconstituents present in the *Morinda tinctoria* leaf extract. *M. tinctoria* leaf acetonic extract‐mediated AgNPs were used to assess the larvicidal potential and it shows high mortality values of 50% against *C. quinquefasciatus* [139]. AgNPs mediated by *M. Tinctoria* possess a promising larvicidal activity against dengue vector *Aedes aegypti* at the stage of 3rd instar larvae and recorded 50% lethal concentration of leaf extract and *M. tinctoria*‐mediated AgNPs showed that LC_50_ was found to be 11.716 ppm. Terpenoids have high potential in converting the aldehyde to carboxylic acid group in metal ion. The size and stability of AgNPs was considered to be an important potential to control mosquitoes in the stage of larvae. Hence, AgNPs act as a promising insecticide agent, causing the desired mortality against the target organism [140]. The physiological explanation for the high lethality of AgNPs produced by using plant extract is still unknown. It is now hypothesised that the AgNPs have the capacity to permeate the invertebral shell and then into the insect's cell, wherein they bind with the macromolecules like proteins, and DNA thereby changing the structure and their functionality. Surprisingly, plant‐mediated AgNPs are harmful to several mosquito larvae and have no effect on beneficial species and the cause of this behaviour is unknown. It is critical to emphasise that more research studies are necessary to confirm the processes through which AgNPs cause harm to their designated target. This evidence will indeed be essential in determining the usage of nanoparticles for vector control, which could have any unanticipated serious impact on the environment and human health.

### 7.10 Anti‐oxidant activity

The oxidative process inside the human body produces oxidative stress, which is reactive oxygen species. The human body possesses several antioxidative stress defensive systems, such as enzymes and chemicals. Reactive oxygen species (ROS) are produced by cells during immune function and respiration which are reactive species. If their concentration is higher, it results in coupling with the biomolecules. This form of interaction is fatal, tends to result in cardiovascular disorders, cancer, ageing, atherosclerosis, and inflammatory diseases [141]. Table 4 shows the antioxidant activity of various medicinal plants‐mediated synthesised AgNPs. *Taraxacum officinale* (leaf)‐mediated AgNPs exhibits the potential antioxidant (68.2% at 100 μg/ml) and it is beneficial to create or co‐formulat new pharmaceutical products [146]. AgNPs synthesised from *Musa paradisica* bract extract has been portrayed as an effective radical scavenger and the extract acts as both reducing and capping agent. Because of their higher dispersion of AgNPs, the synthesised AgNPs effectively inhibit the growth of phytopathogens and also has better stable colloidal nanoparticles. Furthermore, they suppress the free radical production to prevent cell damage. The AgNPs solution has proton‐donating properties, it may act as a free radical scavenger. Hence, it can be used as a potential application in the pharmaceutical and agriculture fields [147]. AgNPs produced using *Artemisia quttensis* extract was rapid, cost effective, simple, and has good stability with the promising antiradical potential, which can be used in nutraceutical and pharmaceutical industries [148]. It could be noted that alkaloids, glycosides, flavonoids, saponins, carbohydrates, phenolic compounds, and tannins found in the plant extract are indeed a valuable root for synthesising stable AgNPs in a short period of time, and they already play a key role in the reduction and stabilisation actions that contribute to AgNPs production.

**TABLE 4:** Antioxidant activity of various medicinal plants and its parts mediated synthesised silver nanoparticles (AgNPs) with their morphological distribution

S. No	Plant name (part)	UV‐vis SPR (nm)	Size (nm)	Morphology	Possible biomolecules involved in the reduction and stabilisation	Applications	Reference
1	*Acacia nilotica* (leaf)	300	10–50 (SEM)	Spherical, uniform distribution	Proteins and phenolic compounds arising from carbonyl and OH stretching	Antioxidant activity	[71]
2	*Cassia angustifolia* (flowers)	452	10–80 (SEM)	Spherical	Phenols, carbonyls, nitro compounds, aromatics, akane compounds, alkyl halides	Antioxidant activity	[142]
3	*Cinnamomum zeylanicum* (bark)	425	30–150	‐	‐	Antioxidant activity	[143]
4	*Phyllanthus amarus* (leaf)	404	‐	‐	Hydroxyl, and amine	Antioxidant activity	[91]
5	*Solanum torvum* (fruit)	430	5–50 (SEM)	Spherical	Hydroxyl/amine	Antioxidant activity	[94]
6	*Taraxacum officinale* (leaf)	435	15	Spherical	Aromatic amines, alcohols, and carbonyl group	Antioxidant activity	[144]
7	*Tinospora cordifolia* (leaves)	430	30 (XRD)	Spherical	Alkanes, lipids, and proteins	Antioxidant activity	[145]

### 7.11 Anti‐cancer activity

Cancer is a life‐threatening illness that leads to deaths across the globe [149]. Cancer is a multifactorial disease caused by a complex combination of genetic and environmental factors. However, awareness of cancer's genetic, biochemical, and cellular origin can provide new therapeutic goals and strategies. Without causing unnecessary irreversible damage to healthy tissues and cells, many anticancer drugs enter their target site at inadequate concentration and effectively exercise the pharmacological impact [150, 151, 152]. Several chemotherapy medicines are now being employed to treat various types of tumours and the side effects are substantial, and intravenous infusion delivery of chemotherapeutics is a time‐consuming procedure. Several research labs have employed a variety of cell lines to investigate the possibilities of discovering a novel cancer fighting agent.

Nanoparticle‐mediated therapy is the best and alternative therapeutic strategy in cancer therapy. NPs have been employed as medication delivery system because of their potential to target specific sick cells or tumour tissues by encapsulating the therapeutic drug in the nanoparticles [153, 154, 155]. Even though several nanoparticle‐mediated techniques have been developed, physicians and nanotechnologists are facing a massive challenge in developing specialised compositions to specifically target individual cancer, due to the variation of the tumour and its surroundings. Novel nanoparticles are used in single platform‐based techniques to improve overall specificity, less toxicity, biocompatibility, and improved efficiency while overcoming the drawbacks of standard chemotherapy. The obstacles and limits of employing nanoparticles for cancer treatment, such as physiological limitations and low carrying capacity should be addressed [153]. AgNPs communicate with the mitochondria and interrupt the role of the cellular electron transfer system, resulting in an increased amount of ROS. As a result, ROS's oxidative stress was considered the key mechanism of toxicity against cells by AgNPs. As a result, AgNPs might easily reach the cells and interact with cell constituents due to their huge surface area, which will disrupt the pathway of cellular signalling.

Table 5 shows the Anticancer activity of various medicinal plant‐mediated synthesised AgNPs. The AgNPs synthesised using *Vitex negundo* (leaf) showed activity against the HCT15 line of human colon cancer growth inhibition. This study dependent inhibition, which diminishes cell viability. The shrunk, restricted, treated, and healthy regulated cells are shown by cytomorphology observation. Apoptotic shifts and nuclear condensation are investigated using propidium iodide staining and DNA fragmentation by single‐cell gel electrophoresis. This result recorded that biosynthesised AgNPs at IC50 of 20 μg/ml inhibited the multiplication of colon cell line HCTL5 (human), stopped the G0/G1 process, observed decreased DNA synthesis, and induced apoptosis [166]. AgNPs synthesised by *Solanum trilobatum* unripe fruit extract was used to check the cytotoxicity against breast cancer MCF7 cell line and showed promising activity. These findings suggested that AgNPs triggered the death of the cell line, and the mitochondrial pathway could be involved. Mitochondria are essential signalling centres during apoptosis, and several apoptosis regulators can cause or inhibit the damage of mitochondrial integrity [167]. *Punica granatum* (leaf)‐mediated AgNPs were stabilised and reduced by the phytochemicals present in the leaf extract. AgNPs showed 50% cytotoxicity, at 100 μg/ml [168]. AgNPs were synthesised using extracts of different plant origins such as *Cucurbita maxima* (petals), *Acorus calamus* (rhizome), *Taraxacum officinale* (leaves), *Moringa oleifera* (leaves), and *Withania coagulans* (leaves). Among these extract‐mediated AgNPs, *A. calamus* (rhizome)‐mediated AgNPs exhibited enhanced activity against A431 carcinoma cells with the IC50 value 78.58 ± 2.7 μg/ml. Besides AgNPs produced using rhizome of *A. calamus* has superior activity than AgNPs mediated with petal and leaf of *A. calamus* [169]. AgNPs used with *Taraxacum officinale* [144] leaf extracts are active against human liver cancer cells (HepG2). The anti‐tumour potential of *Taraxacum officinale‐*mediated AgNPs was comparable to commercial anticancer medicines, which is significant. The leaves from the endangered herb *Withania coagulans*‐mediated AgNPs aid in the active tackling of cervical cancer, hyper triploid cell lines (SiHa). The observed apoptosis was at the point of 13.74 g/ml (IC50) AgNPs concentration [170].

**TABLE 5:** Anticancer activity of various medicinal plants and its parts mediated synthesised Silver nanoparticles (AgNPs) with their morphological distribution

S. No	Plant name (part)	UV‐vis SPR (nm)	Size (nm)	Morphology	Possible constituents involved in the reduction and stabilisation	Applications	Reference
1	*Elettaria cardamomum* (seed)	456	‐	‐	Alcohols, carboxylic acid, ethers, esters, aliphatic amines.	Invitro cytotoxicity activity (Hep‐2 cell line)	[78]
2	*Cassia angustifolia* (flowers)	452	10–80 (SEM)	Spherical	Phenols, carbonyls, nitro compounds, aromatics, alkane compounds, alkyl halides	Cytotoxicity (human breast cell‐MCF‐7)	[142]
3	*Eclipta alba* (leaf)	433	310–400 (SEM)	Monodispersed spherical	Alcohols, phenols, secondary amines and amides,	Cytotoxicity against‐RAW254.7, MCF‐7 and Caco‐2 cells	[156]
4	*Embelia ribes* (berry)	430	30 (TEM)	Spherical	Hydroxyl, carboxyl, and aliphatic amines	Anticancer against MCF‐7	[140]
5	*Erythrina indica* (root)	438	20–118 (HR‐TEM)	Spherical	Alkynyl, amide, proteins, alkaloids, and phenols	Cytotoxic effect on breast and lung cancer cell	[157]
6	*Madhuca longifolia* (leaves)	420	18–24 (TEM)	Spherical	Hirsutrin, isorhamnetin‐3‐glucoside, bilobalide, myricitrin, and esculin	Anticancer activity against breast cancer cell line	[158]
7	*Melia azedarach* (leaves)	436	78 (SEM, DLS)	Spherical	Tannic acid	Cytotoxicity against HeLa cell lines	[159]
8	*Mukia maderaspatna* (leaves)	380–450	20–50 (TEM)	Irregular	Carbohydrates, alkaloids, flavonoids, tannins, phenols, saponins, glycosides, triterpenes.	Cytotoxicity against MCF 7 breast cancer cell lines	[160]
9	*Plumbago indica* (root)	420	50–70 (TEM)	Spherical	Plumbagin (natural naphthoquinone), flavonoids, tannins, alkaloids, saponins, and phenolic compounds	Cytotoxicity against DLA cells	[161]
	*Solanum trilobatum* (fruit)	432	12.50–41.90 (SEM)	Spherical	Hydroxyl/amines	Anticancer activity against human breast cancer cell line MCF 7	[95]
10	*Syzygium aromaticum* (cloves)	441	5–40 (TEM)	Spherical	Proteins	Anticancer activities against MCF 7 breast and A549 lung cell lines	[162]
11	*Taraxacum officinale* (leaf)	435	15 (particle size histogram)	Spherical	Aromatic amines, alcohols, and carbonyl	Anticancer potential against human liver cancer cells HepG2	[144]
12	*Adhathoda vasica* (leaf)	380–430, 438, 420	11.5 (TEM), 21.1–29.1 (SEM)	Poly‐dispersed spherical	Carboxylic acid, and amine groups	Anticancer activity on Hep‐G2 cell lines	[163, 164, 165]

AgNPs possibly provide the ideal surface in mitochondria, where Ag^+^ can bind with the DNA, protein and interfere with their functions [171]. Moreover, ROS generation and oxidative stress arise as an initial event that leads to nanoparticle‐induced toxicity [172]. The cytotoxicity is triggered, because of significantly improved mitochondrial function [173]. AgNPs changed cell shape, reduction in cell stability, physiological performance, enhanced oxidative stress, which resulted in mitochondrial dysfunction [174]. Biologically generated AgNPs are designed to modify cancer cell shape, which is a precursor to apoptosis. Transmitted light microscopy could be used to detect apoptosis by looking at morphological changes in cells. The mechanism of AgNP‐induced cell apoptosis is the next fascinating part of silver research. AgNPs have been shown to be effective in absorbing cytosolic proteins on their surface, which might impact the performance of cellular constituents, as well as regulating expression of genes and pro‐inflammatory cytokines [175]. Autophagy could have a dual feature: at low concentrations, this could help cells to survive, while at high amounts, this could kill them [176]. Autophagy inhibitors improved AgNP‐induced apoptosis in cancer cells. The increased intracellular ion concentration boosts the generation of ROS. The lysosomes destroy endocytosed AgNPs, and the leakage of Ag^+^ ions into the cytoplasm causes cellular injury [177]. The cytotoxicity and genotoxicity of AgNPs are influenced by the processing period used to synthesise them [178]. AgNPs not only caused oxidative stress, but also revealed the impact of glucose content in the media on energy production. All these investigations demonstrated that AgNPs could cause apoptosis through a variety of mechanisms, including increased lactate dehydrogenase release, overexpression of apoptosis and autophagy genes, mitochondrial malfunction, caspase stimulation, and damage to DNA.

### 7.12 Silver nanoparticles for drug delivery system

Drug pharmaceuticals and pharmacodynamics are just as essential as fundamental therapeutic benefits of medicine. Nanoparticles have gained a great deal of interest when it comes to the implementation and production of unique and advanced drug delivery methods [179]. It is important to assess the techniques used during the distribution of the specified pharmaceutical element in human or animal species to provide certain therapeutic efficacy. The combination of chemical units containing AgNPs were effectively selected for acquiring novel and successful drug delivery systems adaptable to thermal, pH modifications to target inflammation, highly contagious, and viable features for nanoscale‐derived health care settings [180]. A good activable and adjustable nano system for drug delivery applications must be simple to build with easily accessible components and have outstanding reactivity. The drug delivery technology must also provide appropriate flexible drug concentration and discharging characteristics. It should also provide maximum therapeutic efficiency at concentrations lower than the main compounds which minimises the side effects [181]. AgNPs have received a lot of interest in this area, and they have been proven to be powerful anti‐tumour drug delivery systems, operating as active or passive nanocarriers for anticancer medicines [52]. AgNPs could be used as vaccines and medication vehicles for cell or tissue targeting that is precise and specific. AgNPs has excellent optical characteristics; recent advances in AgNPs bioactivity and durability through surface modification strongly suggest silver‐based nanostructured frameworks as specific, selective, and flexible alternatives for drug delivery.

### 7.13 Silver nanoparticles for wound healing

Wound infections are serious therapeutic problems that have a significant influence on patient suffering and fatality, as well as substantial economic consequences. Controlling wound sprouting and clinical illness is a difficult but a necessary task. Although the skin is the largest and most advanced component of the human body, it is susceptible to various hazardous environmental stimulation. Based on the level of trauma, physically and chemically created cutaneous wounds could drastically disrupt skin functional stability at various stages, resulting in lifelong impairment or even mortality [182, 183]. Wound infections induced by opportunistic bacterial pathogens have become a major concern in healthcare settings. Rapid tissue regeneration, combined with maximum functionality reconstruction and minimum fibrous tissue development, is the optimum trend and perfect prerequisite for infected wound therapy. Coagulation, swelling, cellular proliferation, and tissue remodelling are considered to be the components of wound healing process [184, 185]. For the innovative and successful management of certain illnesses, silver‐based chemicals and materials are adopted. AgNPs has a broad range of effective bactericidal activity due to its inherent physicochemical properties and biological characteristics.

The production of AgNPs from *A. solanacea* has potential wound healing capacity and will act as an effective biomedical application. AgNPs synthesised from an aqueous extract of *Earliella scabrosa* showed good stability and showed significant would healing activity with 68.58% of wound closure. Cell migration and proliferation plays an important role in the healing process by initiating the repairing process [186]. The production of silver nanoparticles mediated by the potential herb *Onosma dichroantha* proposed the development of bactericides with a broad range of applications in treating injuries and burn wounds [187]. Ionization of silver is essential to provide certain antibacterial actions in physiological settings. Silver ions binds with structural and structural proteins when they enter the cells. AgNPs which are applied in absorbent wound dressings, could kill the germs present in extrude. Diabetic wounds could be associated by a variety of alternative ailments; the use of AgNPs and Ag^+^ carriers is also a beneficial technique for diabetic wound healing process. AgNPs could improve the diabetic individual in the initial phases of wound healing treatment [188]. AgNPs have effective and increased antibacterial activities, as well as significant attention in their use in wound treatment and medical device coatings, their biocompatibility and toxicity should be completely studied.

### 7.14 Other applications

**FIGURE 8:**
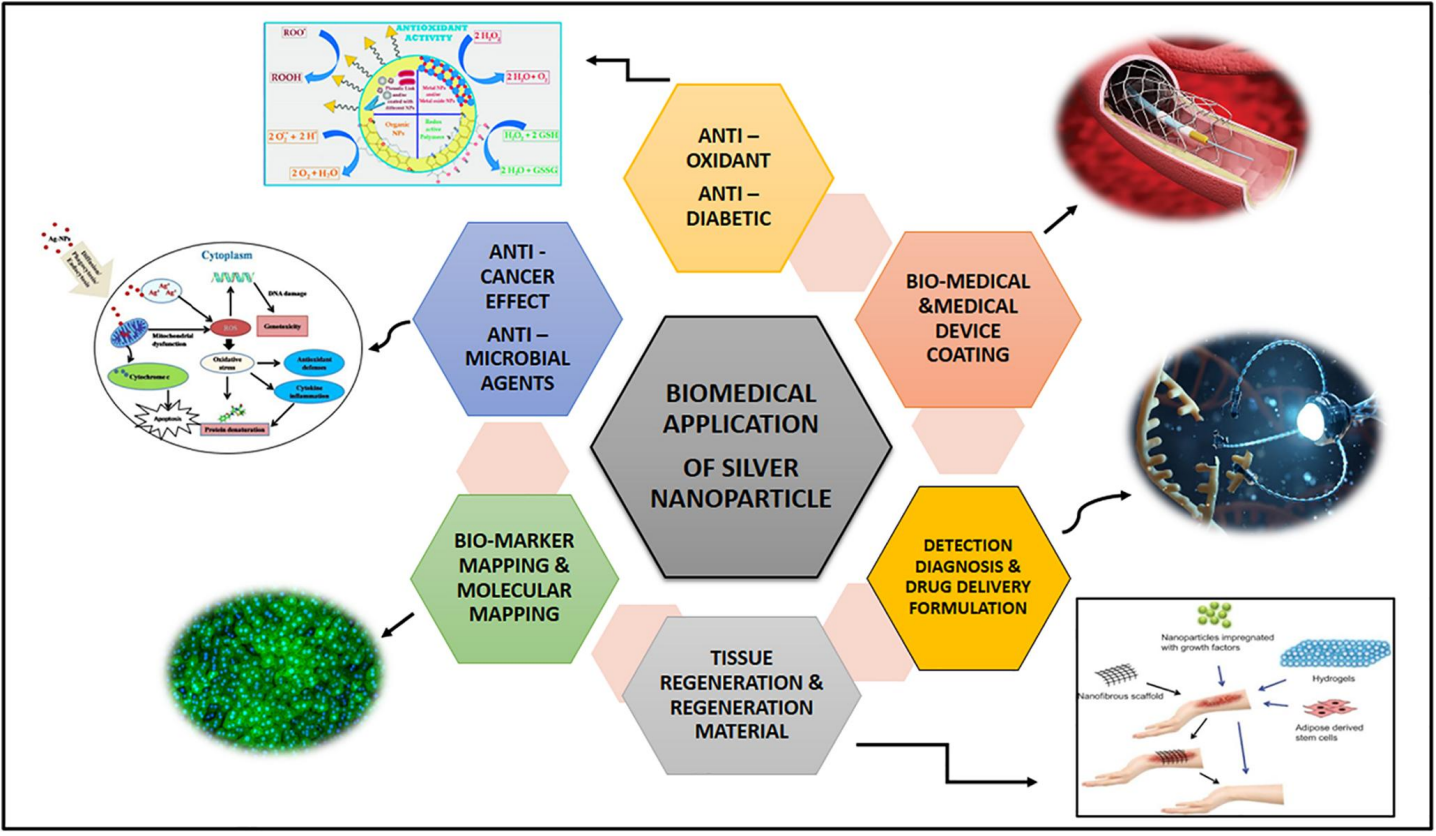
Overall view of biomedical application of silver nanoparticles (AgNPs)

The use of nanoparticles in future is extremely promising, as the excessive spread of microbial contaminations is currently a major danger all over the world. Nanoelectronics, molecular imaging, diagnostics, and biomedicine are just a few of the applications of AgNPs in nanomedicine. The use of an increased electromagnetic field on and near the surface of AgNPs is the basis for these novel applications [189]. AgNPs have very sensitivity NPs probe that can be used to target and image the DNA, small molecules, proteins, cells, tissues, and even tumours. AgNPs with a higher plasmon resonance has indeed been frequently utilised in imaging systems, especially for cellular imaging with contrasting compounds stabilised to AgNPs by surface treatment [190]. In bedridden patients, central venous catheters (CVC) are typically intended to give accessibility for intravenous fluid delivery, haemodynamic assessment, medication delivery channels, and nutritional assistance [190]. Nonetheless, these medical devices represent a significant cause of hospital‐acquired illnesses, and they are classified as an excellent class of devices sensitive to bacterial contamination and colonisation [191]. Under the right humidity and temperature circumstances, the human eye is an important system with amazing vascularisation and communication, which can be readily contaminated by microbes [192]. AgNPs showed promising activity in the production of novel and performance‐enhancing treatments for eye‐related bacterial infections. AgNPs encapsulated with calcium signals minimised retinal cell damage and might be used for retinal imaging [193]. *Musa paradisiaca* bract extract‐mediated synthesised AgNPs exhibited a higher inhibitory zone against fungal pathogens and their catalytical efficacy showed it to be a promising nano‐catalyst to degrade methyl orange to hydrazine derivative, methylene blue to leuco‐methylene blue, and o‐nitro phenol to o‐ aminophenol [147]. AgNPs were shown to be effective as new nanostructured systems for cancer diagnosis and therapy. AgNPs' wide biocompatibility renders them as potential drugs not just for anti‐infective methods, but also for crucial tumour and MDR resistance initiatives. Other applications of AgNPs were shown in Table 6. The overall view of other biomedical applications is shown in Figure 8.

**TABLE 6:** Other biomedical applications of various medicinal plants and its parts mediated synthesised silver nanoparticles (AgNPs) with their morphological distribution

S.No	Plant name (part)	UV‐vis SPR (nm)	Size (nm)	Morphology	Possible constituents involved in the reduction and stabilisation	Applications	Reference
1	*Adhathoda vasica* (leaf)	380–430, 438, 420	11.5 (TEM), 21.1–29.1 (SEM), 21 (XRD)	Poly‐dispersed spherical	Carboxylic acid, and amine groups	Bio‐fabrication	[163]
2	*Aegle marmelos (fruit)*	423	34.7 (AFM)	Spherical	Flavonoids, terpenoids, protein, alkyl halides, and alkenes	Antimicro fouling activity	[194]
3	*Centella asiatica* (leaf)	410	15.11 ± 2.09	Spherical	Amide	Gelatin based nano composite film	[195]
4	*Curcuma longa (leaf)*	350	15–40 (HR‐TEM)	Spherical	Carboxylic, hydroxyl, alkenes, and carbonyl	Coating on the cotton fabric for antimicrobial and wound healing activity	[196]
5	*Mangifera indica* (leaves)	393	32.4 (XRD)	Crystalline	‐	Dental restoration and antibacterial activity against *E. coli and S. aureus*	[135]
6	*Mentha piperita* (leaf)	‐	35 (TEM)	Spherical	‐	Effect on acetyl cholinesterase activity	[197]
7	*Momordica charantia* (fruit)	400	78.5–100 (SEM, TEM)	Irregular	Amine, alkane, carboxylic acids, ester, and alkynes	Anthelmintic activity	[198]
8	*Momordica charantia* (fruit)	410	5–20 (TEM)	Spherical	Hydroxyl, carbonyl, and alkane	Wound dressing application	[199]
9	*Tribulus terrestris* (leaf)	436	15–40 (XRD)	Spherical	Polysaccharide, amide, protein, flavonoid, and polyphenol	Catalytic activity	[200]
10	*Zingiber officinale* (rhizome)	424	6–20 (particle size analyser)	Mono dispersed uniform shape	Alkanes, alkaloids, and flavonoids	Blood compatibility	[201]
11	*Eclipta protrata* (leaf)	420	45 (TEM)	Triangles, Pentagons and Hexagons	Fatty acids, carbonyl group, flavanones, amide 1 band, and proteins	Larvicidal activity against filariasis and malaria vectors	[140]
12	*Morinda tinctoria* (leaf)	409	60–95 (AFM)	Spherical	Carboxylic, alkyl halide, hydroxyl, amide, alkane, alkene, flavonoids, triterpenoids, polyphenols, and enzymes	Larvicidal activity against *Aedes aegypti L*.	[139]
13	*Morinda tinctoria* (leaf)	409	60–95 (AFM)	Spherical	Carboxylic acid, alkyl halide group, hydroxyl, benzene, flavonoids, triterpenoids, and polyphenols	Larvicidal activity against *Culex quinquefasciatus*	[202]
14	*Mucuna pruriens* (seeds)	437	10–27 (TEM)	Spherical, Triangle, Oval and Circular	Amide amino group, amine, alkane, proteins, and enzymes	‐	[203]
15	*Mukia maderspatana* (leaves)	427	13–34 (FESEM)	Irregular	Amine, and alcohol	Larvicidal activity against *Culex quinquefasciatus and Aedes aegypti*	[204]
16	*Phyllanthus niruri* (leaf)	420	30–60 (SEM)	Spherical	Carbonyl, aliphatic amines, polyphenols, and hydroxyl group	Mosquitocidal property against *Aedes aegypti*	[205]

## 8 POTENTIAL HAZARDOUS EFFECTS OF SILVER NANOPARTICLES

Even though AgNPs have numerous features that make them an ideal candidate for novel and potential medicinal applications, their toxicity has subsequently become an area of attention. AgNPs are commonly advertised as very efficient antibacterial agents that are safe for healthy mammalian cells but the toxicity of AgNPs is connected with their conversion to biological and environmental systems. AgNPs are used in electronic technologies, toys, cleaning products, scientific equipment, and the food processing industry [206]. They may provide hazardous risk to aquatic creatures; widespread usage of AgNPs as antimicrobials and disinfectants may increase bacterial resistance [207]. The toxicity of AgNPs in aquatic species is larger than that in land animals and humans. Ecological toxicity primarily linked to the continuous release of nano‐sized silver, and the chemicals’ impact on marine life is due to their larger dispersion and discharge [208]. AgNPs have the potential to cause considerable oxidative stress to the cellular membrane and organelles including the nucleus, mitochondria, and lysosomes resulting in necrotic or lethal responses. AgNP‐induced oxidative stress could trigger inflammatory reaction, such as the stimulation of innate immunity and an increase in endothelial leakage. AgNPs could cause chromosomal abnormalities, DNA damage, and potential carcinogenicity when inoculated at non‐cytotoxic dosages. Physicochemical characteristics such as dispersion rate, concentration, surface characteristics, size, shape impact the genotoxicity and cytotoxicity of AgNPs [209]. The AgNP‐based materials are primarily used to distribute and describe a wide range of toxicological issues and also to construct the toxicity paradigm when intruding on the biological system.

## 9 ECONOMIC LIMITATIONS

Separating AgNPs from the aqueous suspension is difficult, another constraint is the purity and yield of produced final products. Obtaining high yield is one of the most pressing challenges in biosynthesised AgNPs which could be achieved by altering the synthesis parameters. The primary economic concern is that most research have been conducted at the laboratory level, and there is very little evidence on the application's efficiency on an industrial level. Huge volumes of plant extracts are needed for the manufacturing could be the major hindrance for large‐scale production. Moreover, to produce large quantities of phytochemical, a huge amount of biomass waste will be generated through this synthesis process. Further critical considerations include maintaining maximum yield and durability of AgNPs, where the mechanism causing these features is unknown. Furthermore, there is a lack of consistency in the parameters employed to estimate AgNPs production among research. Similarly, no cost estimation of AgNPs biosynthesis has been explored.

## 10 RESEARCH INTERMISSION/KNOWLEDGE GAP

The green synthesis of AgNPs has become a major research subject in recent decades. The plant extracts have gained importance among a wide variety of ecological resources due to its simple one‐step affordable procedure, environmental friendliness, and chemical safety. Many studies, although, believed that particular possible function group/phytochemicals present in the plant extract were responsible for the synthesis of AgNPs. Yet, hardly any of the articles specify the specific phytoconstituents involved in the reduction of Ag^+^ and stability of nanoparticles rather than the hypothesised reduction mechanism. This could possibly have an impact on their biomedical applications. This is an issue where there is still room for further research on plant‐mediated nanoparticles synthesis that needs to be resolved. By solving this issue, it could assist in the regulation and production of ideal nanoparticle size and shape for numerous applications. It could also assist in determining the toxicity of the individual phytocompounds used in the synthesis process when they come in connection with the environment.

## 11 FUTURE PERSPECTIVES AND CONCLUSION

Silver nanoparticles are a revolutionary way to detect and treat damages to the human body. Because of its versatile application, it is known worldwide. The use of nanoparticles in medicine offers some exciting possibilities and plays a role in making nano‐robots repair at the cellular level. This reduces the damage to the healthy cells in the body, where the earlier detection of diseases is possible. Silver nanoparticles are being studied in depth as nanostructures for novel and improved biomedical applications.

In summary, medicinal plants have been used since the ancient period for medicine. We believe that some compounds present in the plants have healing properties. The current review encapsulates the currently known possible biosynthesis of silver nanoparticles from medicinal plant extracts and provides a dataset that will assist researchers in their future research on green silver nanoparticle synthesis for biomedical applications. Silver nanoparticles are known for their remarkable antimicrobial and anti‐inflammatory properties, widely used in the medical field. However, the toxicity of silver nanoparticles should be known before its usage, and it suggests knowing the applicable concentration of silver so that it can work expertly without tarnishing the individual and environment. Future ambit for silver nanoparticles in the medical field includes focussing the site for cancer therapeutics and fluorescence imaging where the nanoparticles allow for targeted drug delivery, increased bioavailability and sustained drug release in the target tissues, and enhances the stability of the nanoparticles drug. Further work is needed, however, to investigate the mechanism of plant‐mediated AgNPs involved in biomedical applications, which is still unclear. Such research might give a clear picture of the mechanism and effectiveness of silver nanoparticles.

## CONFLICT OF INTEREST

The authors declare that they have no known competing financial interests or personal relationships that could have appeared to influence the work reported in this paper. There is no potential conflict of interest.

## PERMISSION TO REPRODUCE MATERIALS FROM OTHER SOURCES

None.

## Data Availability

Data sharing does not apply to this article as no new data were created or analysed in this study.
